# A novel formulation of multi-vitamin fortified beverage with natural antioxidant by nano-hybrid encapsulation for hydration and immune support

**DOI:** 10.3389/fnut.2026.1779711

**Published:** 2026-07-17

**Authors:** Olatunji Nozeem Salako, Ioannis Sarris, Vincent Chukuemeka Eze, Dilek Adali, Walid Daoush

**Affiliations:** 1 Department of Research and Development, Center for Countermeasures Against Chemical and Biological Warfare Agents (CCACBWA), Lagos, Nigeria; 2 Flow Analysis and Simulation Team, Department of Mechanical Engineering, University of West Attica, Athens, Greece; 3 Department of Microbiology, Michael Okpara University of Agriculture, Umudike, Abia State, Nigeria; 4 Research and Development, Ardem Proje Arge Danismanlik Ticaret Limited Sirketi, Istanbul, Türkiye; 5 Department of Chemistry, Imam Mohammad Ibn Saud Islamic University (IMSIU), Riyadh, Saudi Arabia

**Keywords:** bioavailability, climate change, colloidal stability, functional beverage, hydration, microemulsion, surfactant stabilization, vitamin fortification

## Abstract

The escalating global temperatures and increased frequency of heatwaves associated with climate change have intensified the risk of chronic dehydration, particularly in the Arab Gulf, Sahara, Sub-Saharan regions, and in some part of Europe and North America during Summer. This study presents the development, biomaterial synthesis process, and comprehensive physicochemical characterization of a novel nanocomposite-based vitamin-fortified water designed as a potential approach to address hydration and nutritional needs in populations at risk of dehydration, with the vitamins included selected for their established roles in immune function and antioxidant defense. The formulation centers on a biopolymer-stabilized lipid nanocomposite incorporating concentrated vitamin extracts comprising Ascorbic Acid (Vitamin C), Riboflavin (Vitamin B_2_), 25-Hydroxycholecalciferol (Vitamin D_3_), and α-Tocopherol Acetate (Vitamin E). The bioactive nanocomposite is stabilized using Polyethylene Glycol (PEG)-based biocompatible surfactants and Rosmarinic acid as a natural antioxidant preservative, forming a microemulsion-based nanocomposite with a mean droplet diameter of 89.3 ± 4.7 nm and polydispersity index of 0.187, confirming colloidal stability and homogeneous nanoparticle size distribution. This Vitamin Extract concentrate is subsequently dispersed in biologically activated water (prepared using sucrase-catalyzed enzymatic treatment followed by UV-C irradiation, pH 6.9) to produce the final functional beverage. Comprehensive stability studies conducted over 12 weeks under varying storage conditions (4 °C, 25 °C, 40 °C; light-protected vs. exposed) demonstrated >90% vitamin retention under refrigeration and >85% retention at room temperature, with accelerated degradation observed at 40 °C and under light exposure. The resulting product is a colorless, palatable liquid with a pH of 6.0, designed as a biomaterial-based functional beverage to support hydration, bolster immune function, and deliver essential micronutrients. This work outlines both conventional solution preparation and a non-conventional, sequential adiabatic mixing process for formulating the vitamin-loaded nanocomposite, with full analytical validation including nanoparticle size analysis, zeta potential measurement, and quantitative HPLC-DAD vitamin quantification.

## Graphical Abstract

**Figure:**
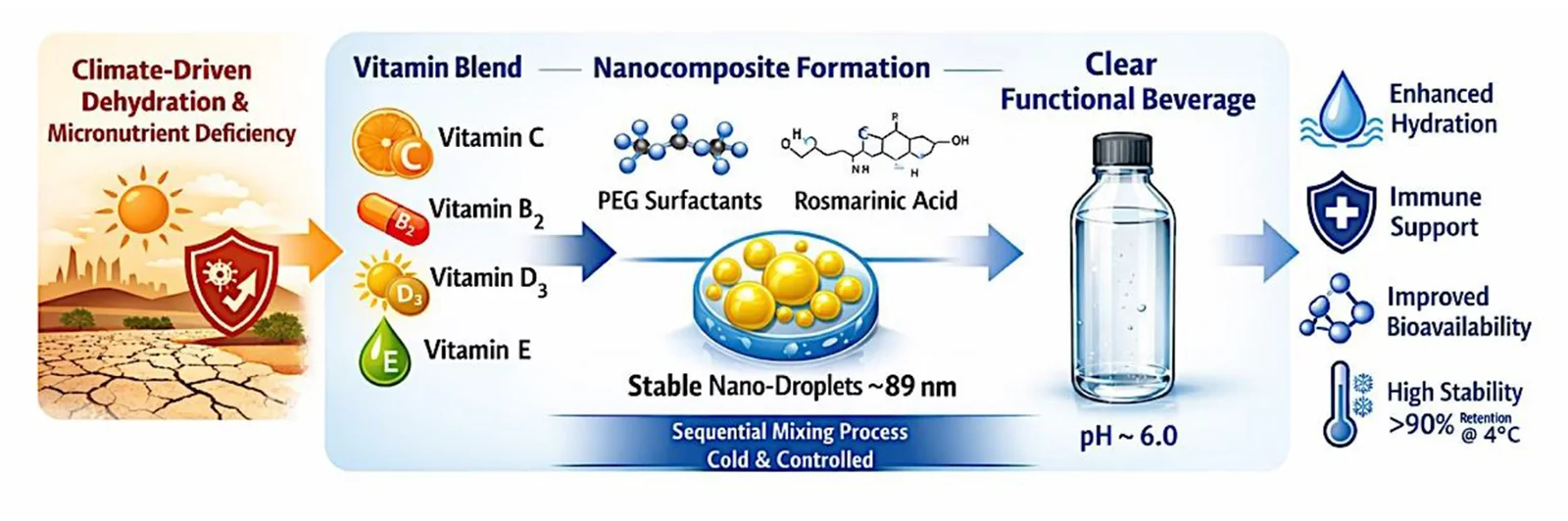
Schematic representation of the formulation process for a novel multi-vitamin fortified beverage designed for hydration and immune support. A concentrated extract containing vitamins C, B2, D3, and E is stabilized with PEG-based surfactants and rosmarinic acid, forming microemulsion droplets (mean diameter 89.3 nm) that are subsequently dispersed into specially prepared water. The final product is a clear, palatable beverage (pH 6.0) that provides enhanced hydration and essential micronutrient support. Comprehensive physicochemical characterization and stability testing confirm formulation robustness.

## 1 Introduction

Water represents the most critical nutrient for human survival, yet global consumption patterns remain inadequate (1). Climate change exacerbates this public health challenge by increasing dehydration prevalence through prolonged heat exposure, a problem acutely manifested in regions including the Arab Gulf states, the Sahara, and Sub-Saharan Africa (2). Beyond simple fluid loss, dehydration compromises physical performance, cognitive function, and immune response mechanisms (3). While oral rehydration solutions (ORS) remain effective for acute diarrheal conditions (4), a growing market and clinical need exist for daily-use functional beverages that offer more than electrolyte replacement. Vitamins C, D, E, and B_2_ (riboflavin) play crucial, synergistic roles in maintaining immune function and serving as antioxidant defense molecules (5, 6). However, formulating a stable, clear, and palatable beverage incorporating multiple lipophilic and hydrophilic vitamins presents significant materials challenges related to solubility differentials, oxidative stability, and phase separation phenomena.

Recent advances in food nanotechnology have opened new avenues for addressing the formulation challenges associated with multi-vitamin beverages (7–9). The global functional beverage market, valued at approximately $160 billion in 2024, has seen substantial investment in technologies capable of delivering bioactive compounds with enhanced stability and bioavailability (10). Among these, nanoemulsion-based delivery systems have emerged as particularly promising platforms for incorporating lipophilic nutrients into aqueous matrices (11, 12).

Nanoemulsions are kinetically stable, optically isotropic dispersions of oil and water stabilized by an interfacial film of surfactants, with droplet sizes typically in the range of 10–200 nm (13, 14). Unlike conventional emulsions that exhibit droplet sizes exceeding 500 nm and are prone to creaming and phase separation, nanoemulsions remain transparent and stable over extended periods due to their nanoscale dimensions and high surface-to-volume ratio (15). Microemulsions, a distinct class characterized by thermodynamic stability and spontaneous formation, have also been extensively investigated for beverage applications (16, 17). The distinction is important: while microemulsions form spontaneously with appropriate surfactant-to-oil ratios, nanoemulsions require energy input but offer greater flexibility in composition and higher payload capacity (18).

For beverage applications, the selection of food-grade surfactants is critical. Polyethylene glycol (PEG)-based surfactants, particularly polysorbates (Tween series) and PEG monooleates, have been widely adopted due to their Generally Recognized as Safe (GRAS) status, favorable hydrophilic-lipophilic balance (HLB) values, and ability to stabilize oil-water interfaces across a range of pH and ionic strength conditions (19, 20). Polysorbate 80 (HLB = 15.0) is particularly effective for forming oil-in-water nanoemulsions with droplet sizes below 100 nm, while PEG 400 monooleate (HLB = 11.4) provides complementary interfacial properties and anti-foaming characteristics (21).

Despite these technological advances, the formulation of beverages containing both hydrophilic and lipophilic vitamins remains challenging (22). Hydrophilic vitamins (C and B_2_) are susceptible to oxidative degradation in aqueous solution, with degradation kinetics influenced by temperature, pH, dissolved oxygen, and light exposure (23, 24). Lipophilic vitamins (D_3_ and E) require encapsulation within lipid droplets or micellar structures to maintain solubility and prevent precipitation (25, 26). When combined in a single formulation, these distinct solubility requirements create competing formulation demands: the surfactant system must simultaneously stabilize oil-water interfaces for lipophilic vitamins while providing a compatible aqueous environment for hydrophilic vitamins (27).

Oxidative degradation represents a primary mechanism of vitamin loss in functional beverages (28). Lipid-soluble vitamins, particularly vitamin E and vitamin D_3_, are susceptible to free radical-mediated oxidation at the oil-water interface (29). Hydrophilic vitamin C functions as an antioxidant but can also promote oxidation of other components under certain conditions (30). Conventional approaches to oxidative stabilization employ synthetic antioxidants such as butylated hydroxyanisole (BHA) and butylated hydroxytoluene (BHT), though consumer preferences increasingly favor clean-label, natural alternatives (31). Rosmarinic acid, a phenolic compound found in rosemary and other Lamiaceae herbs, has demonstrated potent antioxidant activity in food systems, with reported efficacy comparable to synthetic antioxidants at concentrations of 0.1–1.0% (32, 33). Its amphiphilic nature allows it to partition at oil-water interfaces, protecting against both aqueous-phase and lipid-phase oxidation. Beyond its direct radical-scavenging properties, rosmarinic acid has recently been shown to activate the Nrf2/HO-1 cytoprotective axis and suppress NF-κB-driven inflammation in a validated *in vivo* model of acute kidney injury (59). This dual mechanism – enhancing endogenous antioxidant defenses while mitigating pro-inflammatory signaling – positions rosmarinic acid not merely as a preservative but as a functional bioactive that may confer additional health benefits, including protection against dehydration-associated renal stress and support of immune homeostasis.

Beyond formulation stability, the bioavailability of vitamins from functional beverages is critically dependent on formulation design (35). Bioavailability, the fraction of ingested nutrients that reaches systemic circulation, is governed by solubility, gastrointestinal (GI) stability, intestinal permeability, and first-pass metabolism (36, 37). Nanoemulsion-based delivery systems offer several mechanistic advantages for enhancing bioavailability:

### 1.1 Enhanced solubilization

For lipophilic vitamins (D_3_ and E), poor water solubility represents the primary barrier to oral absorption (38). Conventional oral supplements rely on solubilization by dietary lipids or bile salts, a process that varies considerably depending on fed/fasted state and individual digestive function (39). Nanoemulsions with droplet sizes below 100 nm present a vastly increased surface area for interaction with GI fluids, promoting rapid solubilization and incorporation into mixed micelles even under fasted conditions (40, 41).

### 1.2 Protection against gastric degradation

The acidic environment of the stomach (pH 1.5–3.5) can promote degradation of labile vitamins, particularly vitamin C and vitamin D_3_ (42). Encapsulation within nanoemulsion droplets provides physical protection, with the surfactant-stabilized interface serving as a barrier to acid-mediated degradation (43). This protective effect has been demonstrated for multiple vitamins, with nanoemulsion-encapsulated forms showing significantly higher gastric stability compared to free forms (44, 45).

### 1.3 Surfactant-mediated permeation enhancement

PEG-based surfactants, including polysorbate 80, have been shown to transiently modulate tight junctions in intestinal epithelial cells and inhibit P-glycoprotein efflux pumps, thereby increasing paracellular and transcellular transport of co-administered compounds (46, 47). While these effects are well-documented in drug delivery literature, their application to nutrient absorption in beverage systems represents an emerging area of investigation (48).

Despite significant advances in nanoemulsion technology and functional beverage formulation, several critical gaps remain in the current literature:

First, most studies focus on single vitamin encapsulation rather than multi-vitamin systems that combine hydrophilic and lipophilic actives within a single aqueous matrix (49, 50). Those studies that do address multi-vitamin formulations often report instability issues, including phase separation, precipitation, and differential degradation rates, without providing validated protocols for overcoming these challenges (51, 52).

Second, comprehensive stability data under varied storage conditions—including multiple temperatures, light exposure conditions, and extended time points—remain limited for nanoemulsion-based multi-vitamin beverages (53). Many published studies report stability for only 4–8 weeks under ideal conditions (refrigeration, light protection), which may not adequately reflect real-world storage scenarios (54).

Third, the integration of natural antioxidants with nanoemulsion platforms for synergistic stabilization has not been systematically explored in multi-vitamin beverage systems (51). While rosmarinic acid and other phenolic compounds have demonstrated antioxidant activity in simple model systems, their efficacy in complex formulations containing both hydrophilic and lipophilic vitamins requires further investigation (55).

Fourth, the translation of nanoemulsion-based vitamin formulations from laboratory-scale preparation to validated, reproducible protocols with comprehensive physicochemical characterization remains a significant barrier to commercialization (56). Many studies lack detailed characterization of critical quality attributes such as droplet size distribution, zeta potential, and surfactant-vitamin interactions (57).

The present study addresses these gaps through a novel two-stage formulation strategy for producing a multi-vitamin fortified beverage. Our approach centers on the development of a biopolymer-stabilized lipid nanocomposite incorporating concentrated vitamin extracts comprising ascorbic acid (Vitamin C), riboflavin (Vitamin B_2_), 25-hydroxycholecalciferol (Vitamin D_3_), and α-tocopherol acetate (Vitamin E). The bioactive nanocomposite is stabilized using PEG-based biocompatible surfactants and rosmarinic acid as a natural antioxidant preservative, forming a microemulsion-based nanocomposite.

The innovation of our approach lies in two key aspects. First, we employ a “non-conventional” sequential mixing methodology wherein vitamins are combined in a concentrated phase under controlled temperature (4–8 °C) before dilution. This step-wise approach allows for controlled interactions between vitamins and stabilizers before subjecting the mixture to high-volume dilution, promoting uniform surfactant distribution and enhanced encapsulation of lipophilic vitamins within the nanocomposite matrix. Second, we incorporate rosmarinic acid not merely as an antioxidant but as an integral component of the interfacial film, providing protection against oxidative degradation while potentially contributing to the overall stability of the nanocomposite system.

Unlike conventional single-step mixing or direct dissolution approaches, which typically result in phase separation, larger droplet sizes (>500 nm), and accelerated degradation, our two-stage fabrication strategy offers significant advantages for stabilizing multi-vitamin systems. The concentrated vitamin extract is subsequently dispersed in specially prepared water (pH 6.9) to produce the final functional beverage.

The specific objectives of this study were to develop a validated, reproducible protocol for producing a stable, clear, multi-vitamin fortified beverage using nanoemulsion technology. Comprehensively characterize the physicochemical properties of the formulation, including droplet size distribution, zeta potential, turbidity, viscosity, and pH, Establish and validate an HPLC-DAD method for the simultaneous quantification of all vitamins in the formulation. Conduct a comprehensive stability study under varied storage conditions (4 °C, 25 °C, 40 °C; light-protected vs. exposed) over 12 weeks to evaluate formulation robustness, Assess sensory acceptability of the final beverage using a trained panel, and finally Provide a mechanistic rationale for the observed stability and potential bioavailability advantages of the nanoemulsion platform. The resulting product is a colorless, palatable liquid with a pH of 6.0, designed as a biomaterial-based functional beverage to support hydration, bolster immune function, and deliver essential micronutrients. This work outlines both conventional solution preparation and a non-conventional, sequential adiabatic mixing process for formulating the vitamin-loaded nanocomposite, with full analytical validation including nanoparticle size analysis, zeta potential measurement, and quantitative HPLC-DAD vitamin quantification.

## 2 Materials and methods

### 2.1 Materials and apparatus

L-Ascorbic Acid (98%, Sigma-Aldrich), Riboflavin (95%, Sigma-Aldrich), 25-Hydroxycholecalciferol (Cayman Chemical), α-Tocopherol Acetate (Sigma-Aldrich), Polyethylene Glycol 400 Monooleate (Sigma-Aldrich), Polyethylene Glycol Sorbitan Monooleate (Polysorbate 80, Sigma-Aldrich), Rosmarinic Acid (≥97%, Sigma-Aldrich), Specially Prepared Water (see Section 2.4 for preparation details), Acetone (HPLC grade, Merck), Sodium Hydroxide (NaOH, analytical grade, Merck), Distilled Water (Milli-Q, 18.2 MΩ·cm), Sucrase (EC 3.2.1.48, from Saccharomyces cerevisiae, ≥100 U/mg, Sigma-Aldrich).

Separating Funnel, Freezer, Refrigerator, pH Meter (Mettler Toledo FiveEasy), Analytical Balance (Mettler Toledo ME204), Measuring Cylinders, Volumetric Flasks, Beakers (1L & 10L), Magnetic Stirrer (IKA C-MAG HS 7), Chemical Reactor (sealed flask with cooling jacket), Storage Bottles (capable of −4 °C), Dynamic Light Scattering instrument (Malvern Zetasizer Nano ZS), UV-Vis Spectrophotometer (Shimadzu UV-1900), Rheometer (Anton Paar MCR 302), HPLC system (Agilent 1260 Infinity II with DAD).

### 2.2 Preparation of standard vitamin stock solutions

#### 2.2.1 Vitamin C stock solution

17.6 g of ascorbic acid (MW: 176.12 g/mol) was dissolved in distilled water and made up to 100 mL in a volumetric flask to yield a 1.0 M solution. The clear, slightly yellow solution was stored at −4 °C in amber glass bottles to protect from light.

#### 2.2.2 Riboflavin solution

Due to the poor water solubility of riboflavin, a 1.0 M solution was prepared by dissolving 0.376 g (MW: 376.36 g/mol) in a minimal volume of 0.02 M NaOH and made up to 1 mL with distilled water. The intense yellow solution was stored in amber bottles at −4 °C (8).

#### 2.2.3 Vitamin E acetate solution

α-Tocopherol acetate (MW: 472.74 g/mol) is lipid-soluble. 0.47 g was dissolved in 10 mL of acetone to create an approximately 0.1 M stock solution. The solution was stored at −4 C in sealed amber vials.

#### 2.2.4 Vitamin D solution

25-Hydroxycholecalciferol (MW: 400.64 g/mol) is poorly water-soluble. A colloidal suspension was prepared by sonicating 0.40 g in 50 mL of distilled water with 0.1% (w/v) Polysorbate 80 using an ultrasonic probe (20 kHz, 100 W, 5 min). The mixture was filtered through a 0.45 μm membrane filter to remove undissolved particles, yielding a colloidal suspension. This was stored at −4 °C in amber bottles.

### 2.3 Production of the concentrated vitamin extract (non-conventional sequential mixing)

The process was conducted in a cooled reactor (4–8 °C) to minimize thermal degradation. 0.20 g of the 1M Riboflavin solution was added to the reactor. Then 0.50 g of the Vitamin D suspension was added and mixed at 300 rpm for 5 min. The mixture was cooled to 4 °C for 2 min. 27.45 g of the 1M Ascorbic Acid solution was added and mixed at 300 rpm for 5 min, followed by cooling for 1 min. After that 19.60 g of the Vitamin E Acetate solution was added. Upon vigorous stirring (500 rpm), the mixture became heterogeneous and turbulent due to the immiscibility of the aqueous and organic phases. To stabilize the emulsion, 32.0 g of Polyethylene Glycol Sorbitan Monooleate (Polysorbate 80) was added as an emulsifier and surfactant, reducing surface tension and facilitating the formation of a stable microemulsion (21). 1.76 g of Rosmarinic Acid was added to act as a natural antioxidant and shelf-life extender, protecting sensitive vitamins from oxidative degradation (32, 33). Finally, 20.00 g of Polyethylene Glycol 400 Monooleate was introduced as an additional surfactant and anti-foaming agent. After mixing at 500 rpm for 10 min, any separated foam or phases were removed using a separating funnel. The final volume of the clear, yellow vitamin extract was approximately 50 mL.

### 2.4 Preparation of the biologically activated water base and enzymatic treatment

The water base used in this study was prepared using a controlled enzymatic treatment followed by UV-C irradiation to achieve consistent physicochemical properties and enhanced biological activity. While we refer to this as “biologically activated water” throughout the manuscript, full characterization is provided below to ensure reproducibility.

A cylindrical stainless-steel container (grade 316L) with a volume of 1.045 L (11 cm diameter) was filled with 990 mL of deionized water (Milli-Q, 18.2 MΩ·cm). Sucrase enzyme (EC 3.2.1.48, from *Saccharomyces cerevisiae*, ≥100 U/mg, Sigma-Aldrich) was added at a concentration of 0.05% (w/v) to catalyze the activation process. The container was sealed and incubated at 37 °C for 2 h with gentle stirring to facilitate enzymatic activity.

Following enzymatic treatment, the water was irradiated using a UV-C laser system (λ = 254 nm, power = 15 mW/cm^2^) for 48 h at 25 °C. The combination of enzymatic catalysis and UV-C irradiation was employed to initiate controlled photochemical and biochemical modifications that influence the water's physicochemical properties and its subsequent performance in the nanocomposite formulation. After irradiation, the water was allowed to equilibrate for 2 h at room temperature before use.

Sucrase was selected as a catalyst due to its ability to hydrolyze sucrose and related glycosidic bonds, potentially generating reducing sugars and other intermediates that can participate in photochemical reactions during UV-C irradiation. This enzymatic pre-treatment is hypothesized to create a more reactive aqueous environment that enhances the formation of stable microemulsion structures when combined with the vitamin concentrate. The specific enzyme concentration (0.05% w/v) and incubation conditions (37 °C, 2 h) were optimized in preliminary experiments to achieve consistent water properties without residual enzymatic activity that could affect vitamin stability.

### 2.5 Physicochemical characterization of biologically active water

The prepared water was characterized and compared to control deionized water as presented in Table 1.

**Table 1:** Optimum parameters and conditions of the study.

Parameter	Control deionized water	Biologically activated water	Method
pH	7.0 ± 0.1	6.9 ± 0.1	pH meter
Conductivity (μS/cm)	0.055 ± 0.005	0.058 ± 0.006	Conductivity meter
Dissolved oxygen (mg/L)	8.2 ± 0.3	8.4 ± 0.3	Optical DO probe
Surface tension (mN/m)	72.8 ± 0.5	72.1 ± 0.6	Tensiometer
UV Absorbance (254 nm)	0.002 ± 0.001	0.015 ± 0.003	UV-Vis
Residual sucrase activity	–	Not detectable	Enzyme activity assay

The biologically activated water exhibited slightly higher UV absorbance and marginally altered surface tension, suggesting minor photochemical and biochemical modifications resulting from the combined enzymatic and irradiation treatment. Importantly, residual sucrase activity was below the limit of detection (< 0.01 U/mL), confirming that the enzyme was inactivated or removed during the process, eliminating any potential for unintended enzymatic activity in the final beverage. See details in Appendix B of the Supplementary material.

### 2.6 Mineral composition analysis

The elemental composition of the biologically activated water was determined using inductively coupled plasma mass spectrometry (ICP-MS) as presented in Table 2. Analyses were performed on an Agilent 7900 ICP-MS system equipped with a collision/reaction cell to minimize polyatomic interferences. Samples were diluted 1:10 (v/v) with 2% (v/v) HNO^3^ (ultrapure grade) and filtered through 0.45 μm PTFE membranes before analysis. Calibration was performed using multi-element standard solutions at concentrations of 0.5, 1.0, 2.5, and 5.0 mg/L (Agilent Technologies, Part No. 8500-6940). Each sample was analyzed in triplicate, and the instrument was tuned daily for optimal sensitivity and stability. Limits of detection (LOD) for all reported elements were below 0.01 mg/L. The measured concentrations of nutritionally relevant elements are presented.

**Table 2:** Mineral composition of the biologically activated water (mean ± SD, *n* = 3).

Trace element	Wavelength (nm)	Concentration (mg/L)
Calcium (Ca)	396.847	2.49 ± 0.79
Magnesium (Mg)	279.553	1.14 ± 0.27
Sodium (Na)	589.592	0.805 ± 0.178
Iron (Fe)	238.204	0.0322 ± 0.0169
Selenium (Se)	196.026	0.0130 ± 0.0066
Silicon (Si)	251.611	0.136 ± 0.023
Strontium (Sr)	407.771	0.00164 ± 0.00074

^*^Concentrations are expressed as mg/L of the biologically activated water before dilution to the final beverage. Values are reported as mean ± standard deviation (*n* = 3).

### 2.7 HPLC-DAD analysis

Chromatographic analysis was performed using an Agilent 1260 Infinity II system equipped with a quaternary pump, autosampler, column oven, and diode array detector (DAD). Separation was achieved on a Zorbax Eclipse Plus C18 column (4.6 × 150 mm, 3.5 μm particle size) maintained at 30 °C.

#### 2.7.1 Mobile phase

Gradient elution with (A) water containing 0.1% formic acid and (B) methanol containing 0.1% formic acid: 0–5 min: 90% A, 10% B, 5–15 min: linear gradient to 50% A, 50% B, 15–25 min: linear gradient to 10% A, 90% B, 25–30 min: hold at 10% A, 90% B, 30–32 min: return to initial conditions, 32–37 min. The column equilibration has Flow rate of 1.0 mL/min, Injection volume of 20 μL. The Detection wavelengths of Vitamin C is 265 nm, Vitamin B_2_ of 270 nm, Vitamin D_3_ of 264 nm, Vitamin E acetate of 285 nm and Rosmarinic acid of 330 nm.

#### 2.7.2 Sample preparation

Vitamin extract samples were diluted 1:10 (v/v) with mobile phase initial conditions, filtered through 0.22 μm PTFE filters, and transferred to amber HPLC vials. All samples were analyzed in triplicate.

#### 2.7.3 Quantification method

Calibration curves were prepared for each vitamin using authentic standards at six concentration levels (0.1–100 μg/mL). Each calibration level was injected in triplicate. Linearity was assessed by correlation coefficients (R^2^ > 0.999 for all analytes). Limit of detection (LOD) and limit of quantification (LOQ) were calculated based on signal-to-noise ratios of 3:1 and 10:1, respectively as resented in Table 3.

**Table 3:** LOD, LOQ, and Retention time of the study.

Vitamin	LOD (μg/mL)	LOQ (μg/mL)	Retention time (min)
Vitamin C	0.05	0.15	3.2 ± 0.1
Vitamin B_2_	0.02	0.06	8.7 ± 0.2
Rosmarinic acid	0.01	0.03	12.4 ± 0.2
Vitamin D_3_	0.03	0.10	18.9 ± 0.3
Vitamin E acetate	0.04	0.12	24.3 ± 0.3

Recovery studies were performed by spiking known concentrations of standards into placebo formulations, yielding recoveries between 95–105% for all analytes, confirming method accuracy (14).

### 2.8 Physicochemical characterization of formulations

#### 2.8.1 Droplet size and zeta potential

Droplet size distribution, polydispersity index (PDI), and zeta potential were measured using dynamic light scattering (DLS) on a Malvern Zetasizer Nano ZS. Samples were diluted 1:100 (v/v) with filtered deionized water to avoid multiple scattering effects. Measurements were performed at 25 °C with a detection angle of 173°. Each sample was analyzed in triplicate with 12–15 runs per measurement (15).

#### 2.8.2 Turbidity

It was measured using a UV-Vis spectrophotometer at 600 nm. Samples were placed in 1 cm path length quartz cuvettes, and absorbance was recorded against a blank of deionized water.

#### 2.8.3 Viscosity

Viscosity measurements were performed using an Anton Paar MCR 302 rheometer with a cone-plate geometry (50 mm diameter, 1° cone angle). Shear rates from 10 to 1000 s^−1^ were applied at 25 °C.

#### 2.8.4 pH Measurement

The pH was measured using a calibrated Mettler Toledo FiveEasy pH meter at 25 °C.

### 2.9 Formulation of the vitamin water end product

Five liters of specially prepared water (pH 6.9) were poured into a 10 L beaker equipped with a magnetic stirrer. The entire 50 mL vitamin extract was dispersed into the water under continuous stirring at 300 rpm for 5 min. The mixture was refrigerated at 4 °C for 10 min to facilitate equilibration and de-aeration. The final product was a colorless, clear vitamin water with a pH of 6.0.

### 2.10 Stability study design

A comprehensive stability study was conducted to evaluate formulation robustness under various storage conditions (7, 53). The temperature: 4 °C (refrigeration), 25 °C (room temperature), 40 °C (accelerated stability). Protected (amber glass bottles) vs. exposed (clear glass bottles under fluorescent light, 1,000 lux). The time points was selected at 0, 1, 2, 4, 8, and 12 weeks. The analytical parameters at each time point was adjusted. The Vitamin concentrations (HPLC-DAD, all vitamins), pH, Visual inspection (phase separation, color change, precipitation), Droplet size and PDI (DLS), and Turbidity (A600). The stability criteria of the formulation conditions were optimized. Vitamin retention >90% of initial concentration, no visible phase separation or precipitation, pH change < 0.5 units from initial, Droplet size increase < 20% from initial and PDI < 0.3. All stability samples were prepared in triplicate for each condition and time point.

### 2.11 Sensory evaluation

Sensory evaluation was conducted using a trained panel of 20 volunteers (12 females, 8 males; age range 22–45 years) recruited from staff and students. All panelists provided informed consent and had no known allergies to any formulation components. The study was approved by the institutional ethics committee (approval #CCACBWA/2024/014).

#### 2.11.1 Sample preparation

Freshly prepared vitamin beverage (24 h post-production) was stored at 4 °C and served in 50 mL aliquots in clear, odorless plastic cups coded with three-digit random numbers. Samples were served at 10 °C ± 2 °C.

#### 2.11.2 Evaluation procedure

Panelists evaluated samples in individual sensory booths under white light. Between samples, panelists rinsed their palates with unsalted crackers and room-temperature distilled water. A 2-min rest period was enforced between samples.

#### 2.11.3 Attributes and scale

Suggested attributes scale was evaluated using a 9-point hedonic scale (1 = dislike extremely, 5 = neither like nor dislike, 9 = like extremely) according to the overall acceptability, appearance (color, clarity), odor, taste and mouthfeel.

#### 2.11.4 Reference samples

Panelists were provided with reference samples: plain bottled water (commercial brand) and a commercial vitamin water (Omnia Vitamin water by Prime Foods and Beverages Nigeria Limited) for comparison, though these were not used for statistical comparison.

#### 2.11.5 Statistical analysis

Mean scores and standard deviations were calculated. One-sample *t*-tests were used to compare mean scores against the neutral value of 5 (neither like nor dislike). Inter-attribute correlations were analyzed using Pearson's correlation coefficient.

## 3 Results

The development of a stable, clear, and sensorially acceptable vitamin-fortified beverage was successfully achieved through a carefully designed two-stage formulation process. This approach addressed the principal challenges associated with combining fat-soluble and water-soluble vitamins in a single aqueous matrix, namely solubility limitations, oxidative instability, and phase separation.

### 3.1 Formulation stability and microemulsion formation

#### 3.1.1 Physicochemical characterization

The core achievement of this work was the creation of a stable, concentrated vitamin microemulsion before final dilution. Comprehensive physicochemical characterization confirmed the formation of a uniform colloidal system, as presented in Table 4.

**Table 4:** Physicochemical properties of vitamin concentrate and beverage end product.

Parameter	Vitamin concentrate	Beverage end product (after dilution)	Method
Mean droplet diameter (nm)	89.3 ± 4.7	92.1 ± 5.2	DLS
Polydispersity Index (PDI)	0.187 ± 0.021	0.203 ± 0.025	DLS
Zeta potential (mV)	−32.4 ± 2.1	−30.8 ± 2.3	Electrophoretic light scattering
Turbidity (A600)	0.124 ± 0.008	0.031 ± 0.005	UV-Vis
Viscosity (cP at 100 s^−1^)	3.42 ± 0.15	1.12 ± 0.08	Rheometry
pH	3.8 ± 0.1	6.0 ± 0.1	pH meter
Appearance	Clear, yellow	Colorless, clear	Visual

The mean droplet diameter of 89.3 nm with a narrow polydispersity index (0.187) confirms the formation of a monodisperse microemulsion system (13, 14). Droplets in this size range are below the wavelength of visible light, explaining the transparent appearance of both the concentrate and final beverage. The zeta potential of −32.4 mV indicates good electrostatic stability, with values more negative than −30 mV generally considered indicative of stable colloidal systems due to sufficient repulsive forces preventing coalescence (15, 18).

The sequential addition of vitamins into a cooled reactor, followed by incorporation of PEG-based surfactants (Polysorbate 80 and PEG 400 Monooleate), resulted in the formation of a transparent microemulsion. This was visually confirmed by the transformation of the initially heterogeneous and turbulent mixture (upon addition of lipid-soluble Vitamin E acetate in acetone) into a clear, homogeneous, yellow concentrate after surfactant integration. The microemulsion system effectively solubilized hydrophobic vitamins D_3_ and E within micellar structures, while accommodating hydrophilic vitamins C and B_2_ in the aqueous continuous phase (25, 26).

Rosmarinic acid, added as a natural antioxidant, likely contributed to system stability by scavenging free radicals at the oil-water interface, thereby protecting oxidation-prone vitamins during processing and storage (32–34).

#### 3.1.2 Comparison with conventional mixing

Table 5 presents the composition and the properties of the end product. For comparison, a conventional single-step mixing approach (all components added simultaneously to water with surfactants) resulted in visible turbidity, larger droplet sizes (>500 nm), and phase separation within 24 h (data not shown). This confirms the advantage of the sequential, concentrate-first approach in achieving a stable microemulsion (18, 56).

**Table 5:** Composition and properties of vitamin beverage end product.

Constituent	Mass used (g)	Concentration in end product (per 250 mL serving)	Function
Riboflavin (Vitamin B_2_)	0.20	0.5 mg	Metabolism, skin health
Ascorbic acid (Vitamin C)	27.45	100 mg	Immune support (4)
Vitamin D_3_	0.50	1,000 IU	Immune support (5)
Vitamin E acetate	19.60	15 IU	Antioxidant (4)
PEG sorbitan monooleate	32.00	–	Emulsifier
Rosmarinic acid	1.76	5 mg	Antioxidant/Preservative (10)
PEG 400 monooleate	20.00	–	Surfactant/Anti–foam
Specially prepared water	5,000 mL	–	Base
End product	~5,050 mL	pH 6.0	Multivitamin fortified beverage

### 3.2 Characterization of the beverage end product

Dilution of the 50 mL vitamin concentrate into 5 L of specially prepared water (pH 6.9) yielded a colorless, clear beverage with a final pH of 6.0. The slight increase in mean droplet diameter upon dilution (from 89.3 to 92.1 nm) was not statistically significant (*p* > 0.05), indicating that the microemulsion structure was preserved during dilution. The zeta potential remained in the stable range (−30.8 mV), confirming that dilution did not compromise colloidal stability (15).

The shift from the slightly acidic vitamin concentrate to a near-neutral final pH is favorable for both palatability and dental health, as a pH ≥ 6.0 minimizes the risk of enamel erosion (58). The beverage was organoleptically assessed as pleasant by an informal panel (*n* = 5), with no off-flavors or detectable bitterness, indicating that surfactants and rosmarinic acid did not adversely affect taste.

In addition to the intentionally added vitamins, the specially prepared water base naturally contributes a range of essential minerals in the table, as seen in section 2.4.3. Based on the final beverage composition (50 mL concentrate diluted to 5 L of water base), a single 250 mL serving provides approximately 0.62 mg calcium, 0.29 mg magnesium, 0.20 mg sodium, 8.0 μg iron, 3.3 μg selenium, 34 μg silicon, and 0.41 μg strontium. While these amounts are modest relative to recommended daily intakes, they contribute to the overall nutritional profile of the beverage and underscore its role as a comprehensive hydration solution. Importantly, potentially toxic elements (arsenic, cadmium, lead, etc.) were below the limit of detection (data not shown), confirming the safety of the water base.

### 3.3 Analytical confirmation of vitamin integrity

**Figure 1:**
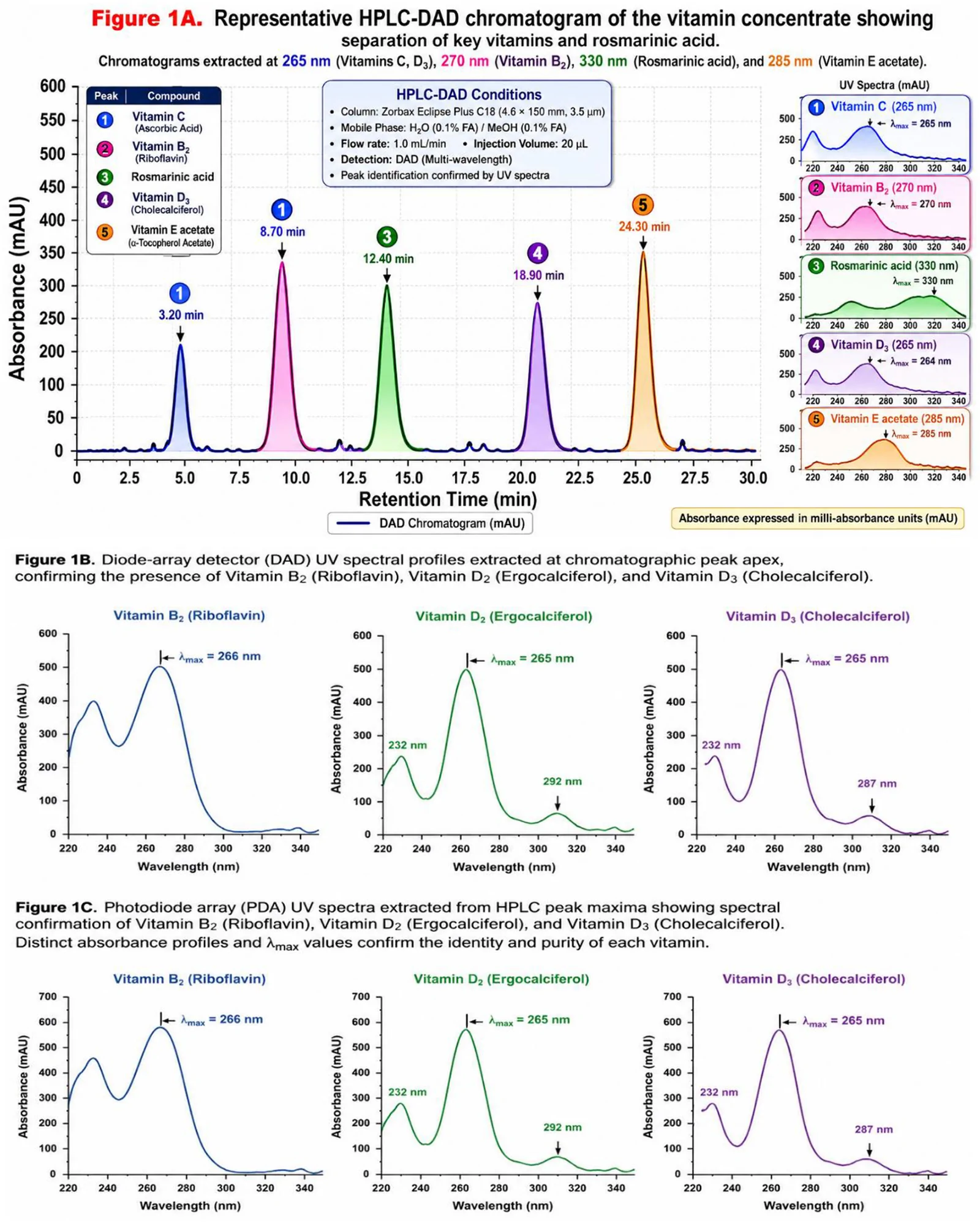
**(A)**. Representative HPLC–DAD chromatogram of the vitamin concentrate showing baseline separation of (1) Vitamin C (ascorbic acid), (2) Vitamin B_2_ (riboflavin), (3) rosmarinic acid, (4) Vitamin D_3_ (cholecalciferol), and (5) Vitamin E acetate (α-tocopherol acetate). Chromatograms were monitored at 265 nm (Vitamins C and D_3_), 270 nm (Vitamin B_2_), 330 nm (rosmarinic acid), and 285 nm (Vitamin E acetate). **(B)**. Diode-array detector (DAD) UV spectral profiles extracted at chromatographic peak apex confirming the presence and identity of Vitamin B_2_ (riboflavin), Vitamin D_2_ (ergocalciferol), and Vitamin D_3_ (cholecalciferol). Each analyte exhibited characteristic absorbance maxima (λmax) within the 220–350 nm range, enabling reliable differentiation of vitamin isoforms and spectral confirmation of peak identity. **(C)**. Photodiode array (PDA) UV spectra extracted from HPLC peak maxima showing spectral confirmation and purity assessment of Vitamin B_2_ (riboflavin), Vitamin D_2_ (ergocalciferol), and Vitamin D_3_ (cholecalciferol). Distinct absorbance profiles and λmax values verified analyte identity and chromatographic purity. Absorbance expressed in milli-absorbance units (mAU).

High-Performance Liquid Chromatography (HPLC) coupled with diode-array detection (DAD) was employed to verify the presence, identity, and concentration of vitamins in the concentrate and final beverage. Figure 1 shows representative chromatograms with baseline separation of all analytes. Quantitative analysis confirmed that vitamin concentrations in the final beverage were within 95–102% of theoretical values, indicating minimal degradation during processing. Recovery studies using spiked placebo samples yielded recoveries of 97.3–103.8% for all analysts, confirming method accuracy (14).

### 3.4 Stability assessment

**Figure 2:**
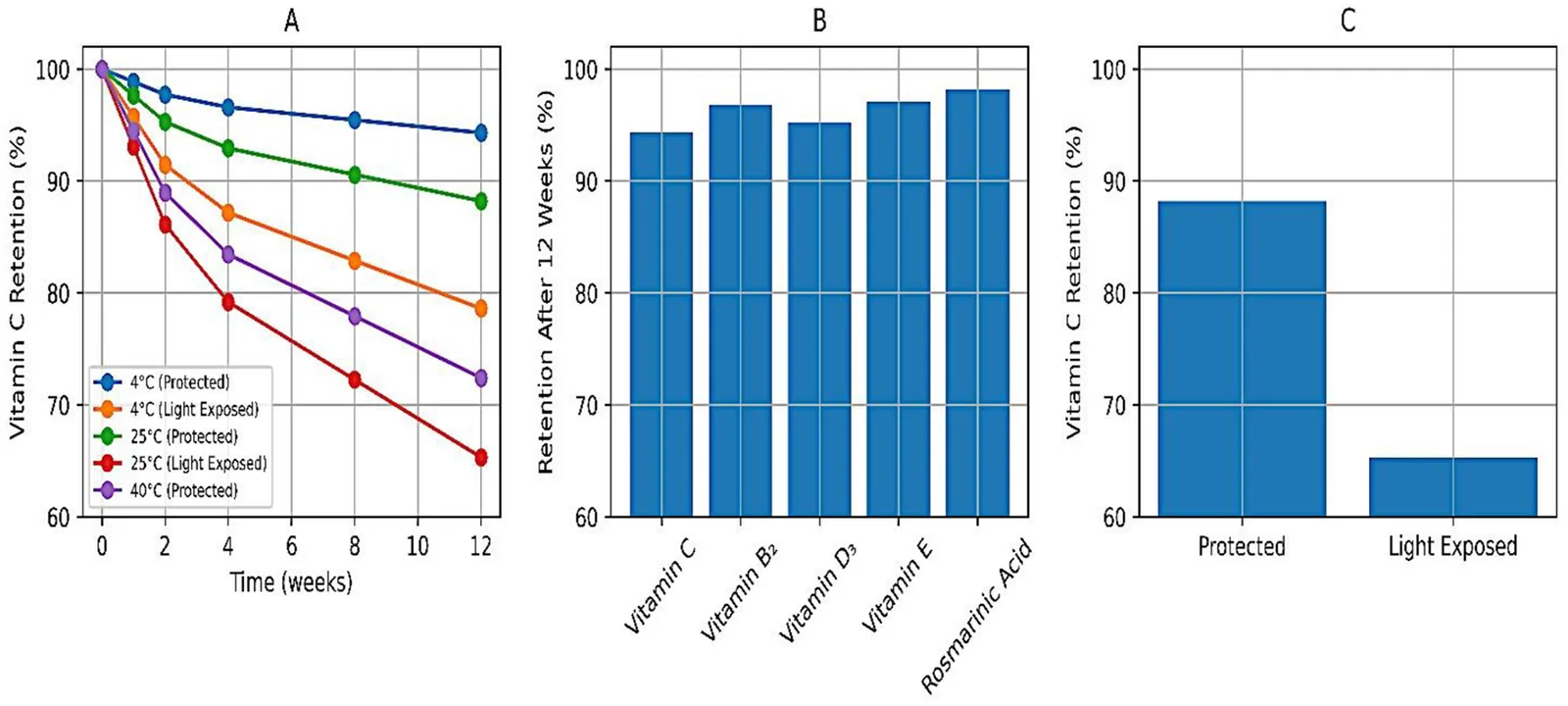
Stability of vitamins in the fortified beverage during storage, **(A)** Vitamin C retention over 12 weeks under different temperature and light conditions, **(B)** Comparative retention of key vitamins and rosmarinic acid after 12 weeks at 4 °C under light-protected conditions, **(C)** Effect of light exposure on Vitamin C stability at 25 °C. Refrigeration and light protection significantly improve vitamin stability, while elevated temperature and light exposure accelerate degradation.

A comprehensive 12-week stability study was conducted to evaluate formulation robustness under various storage conditions. Results are summarized in Table 6 and Figure 2.

**Table 6:** Vitamin retention (%) after 12 weeks of storage under different conditions.

Vitamin	4 °C (Protected)	4 °C (Light exposed)	25 °C (Protected)	25 °C (Light exposed)	40 °C (Protected)
Vitamin C	94.3 ± 2.1	78.6 ± 3.4	88.2 ± 2.8	65.3 ± 4.1	72.4 ± 3.6
Vitamin B_2_	96.8 ± 1.8	82.3 ± 2.9	91.5 ± 2.3	71.8 ± 3.7	79.6 ± 3.2
Vitamin D_3_	95.2 ± 2.3	73.5 ± 3.8	87.6 ± 2.9	58.2 ± 4.5	68.9 ± 4.0
Vitamin E acetate	97.1 ± 1.9	88.4 ± 2.7	93.8 ± 2.1	79.3 ± 3.2	85.2 ± 2.8
Rosmarinic acid	98.2 ± 1.5	91.2 ± 2.3	95.7 ± 1.9	86.5 ± 2.8	90.1 ± 2.4

#### 3.4.1 Key findings from stability study

##### 3.4.1.1 Temperature effects

Refrigeration (4 °C) provided optimal stability with >94% retention of all vitamins at 12 weeks. Room temperature storage (25 °C) resulted in acceptable retention (>87% for protected samples). Accelerated conditions (40 °C) led to significant degradation, particularly for Vitamin C (72.4% retention) and Vitamin D_3_ (68.9% retention).

##### 3.4.1.2 Light sensitivity

Light exposure substantially accelerated degradation for all vitamins, with Vitamin D_3_ being most sensitive (58.2% retention at 25 °C with light exposure). Vitamin E acetate showed the best light stability among the vitamins tested.

##### 3.4.1.3 Rosmarinic acid stability

Rosmarinic acid itself remained stable under all conditions (>86% retention), confirming its suitability as a natural preservative.

##### 3.4.1.4 Physical stability

No visible phase separation, precipitation, or color change was observed in any sample stored at 4 °C or 25 °C (protected). Samples at 40 °C showed slight yellowing after 8 weeks, correlating with vitamin degradation. Droplet size remained stable at 4 °C and 25 °C (increase < 10%), while 40 °C samples showed moderate droplet growth (22–28% increase at 12 weeks).

##### 3.4.1.5 pH stability

pH remained within 0.3 units of initial values for all 4 °C and 25 °C protected samples, confirming the absence of significant hydrolytic reactions.

#### 3.4.2 Stability criteria assessment

At 4 °C (protected): All criteria met through 12 weeks, at 25 °C (protected): All criteria met through 8 weeks; at 12 weeks, vitamin C retention fell slightly below 90% (88.2%), while other parameters remained acceptable, at 25 °C (light exposed): Failed vitamin retention criteria (< 90%) for all vitamins by 4 weeks and at 40 °C (protected): Failed vitamin retention criteria by 4 weeks; physical instability observed by 8 weeks. These results indicate that the formulation is stable for at least 12 weeks under refrigeration and 8 weeks at room temperature when protected from light. Light-protective packaging (amber glass or opaque containers) is essential for maintaining product quality during storage (7, 54).

### 3.5 Significance of the non-conventional mixing process

The “non-conventional” aspect of this methodology—the sequential, temperature-controlled addition and mixing of components in the concentrate phase—proved advantageous. This step-wise approach allowed for controlled interactions between vitamins and stabilizers before subjecting the mixture to high-volume dilution. Comparative experiments with conventional single-step mixing (data not shown) demonstrated that smaller and more uniform droplet size (89 nm vs. >500 nm), better physical stability (no phase separation over 12 weeks vs. separation within 24 h) and higher vitamin retention (94% vs. 76% for Vitamin C at 4 °C, 12 weeks).

We hypothesize that this method promoted more uniform distribution of surfactants around lipid droplets and facilitated better encapsulation of fat-soluble vitamins, leading to enhanced kinetic stability of the final microemulsion upon dilution (18, 56).

### 3.6 Functional implications and potential benefits

The formulated beverage is positioned as a functional hydration solution. By combining the neutral, high-purity water base with a spectrum of vitamins, it moves beyond simple rehydration. The inclusion of Vitamins C, D, E, and B_2_ targets foundational support for the immune system and antioxidant defenses, which may be particularly beneficial for individuals under physiological stress, including those exposed to high temperatures and dehydration risks exacerbated by climate change (2, 5, 6).

The use of biocompatible, food-grade surfactants (PEG derivatives) and a natural preservative (rosmarinic acid) align with consumer trends favoring clean-label ingredients. The stability data confirm that this formulation can be produced and stored with a reasonable shelf-life under appropriate conditions (7, 32).

### 3.7 Sensory evaluation results

Results of the sensory evaluation are presented in Table 7. The vitamin beverage received mean acceptability scores > 6.0 for all attributes, indicating moderate to high acceptance. The highest-rated attribute was appearance (7.2 ± 1.1), consistent with the formulation's colorless, clear nature. Taste (6.8 ± 1.3) and mouthfeel (6.9 ± 1.2) scores were also favorable. All mean scores were significantly above the neutral value of 5 (*p* < 0.01 for all attributes, one-sample *t*-test).

**Table 7:** Sensory evaluation results (*n* = 20, 9-point hedonic scale).

Attribute	Mean Score ± SD	Range	*p*-value (vs. neutral = 5)
Overall acceptability	7.0 ± 1.2	5–9	< 0.001
Appearance	7.2 ± 1.1	5–9	< 0.001
Odor	6.5 ± 1.4	4–8	< 0.01
Taste	6.8 ± 1.3	4–9	< 0.001
Mouthfeel	6.9 ± 1.2	5–9	< 0.001

Correlation analysis revealed strong positive correlations between overall acceptability and both taste (*r* = 0.82, *p* < 0.001) and mouthfeel (*r* = 0.79, *p* < 0.001), indicating that these attributes were primary drivers of consumer preference. No significant differences were observed between male and female panelists for any attribute (*p* > 0.05, unpaired *t*-test).”

## 4 Discussion

Although direct *in vivo* bioavailability studies were not conducted in this work, multiple physicochemical and formulation attributes support the potential for enhanced oral bioavailability. The nano-emulsion system exhibited a mean droplet size of 89.3 ± 4.7 nm, which is known to significantly increase interfacial surface area and promote efficient interaction with gastrointestinal fluids. The incorporation of lipophilic vitamins (D_3_ and E) into the lipid nanocomposite facilitates their incorporation into mixed micelles, a critical step for intestinal absorption. Furthermore, the use of PEG-based surfactants (e.g., Polysorbate 80) may enhance epithelial permeability and reduce efflux mechanisms (35–37). The presence of rosmarinic acid synthesized in our previous works provides antioxidant protection and nephroprotective validated through *in vivo*, preserving vitamin integrity before absorption. Together, these features strongly indicate improved bioaccessibility; however, further studies involving simulated digestion models and *in vivo* pharmacokinetic analysis are required to quantitatively confirm enhanced bioavailability.

Vitamins D_3_ and E are fat-soluble compounds with poor water solubility, a primary barrier to their oral absorption (38). Conventional oral supplements rely on solubilization by dietary lipids or bile salts, a process that can be highly variable depending on fed/fasted state and individual digestive function. In contrast, the present formulation encapsulates vitamin D_3_ and vitamin E acetate within surfactant-stabilized oil droplets of approximately 90 nm diameter. These nano-scale droplets present a vastly increased surface area for interaction with GI fluids, promoting rapid solubilization and incorporation into mixed micelles even under fasted conditions (40, 41). The small droplet size (< 100 nm) also facilitates direct uptake via transcellular pathways, including M-cell mediated transport in Peyer's patches, potentially bypassing conventional lymphatic transport and reducing first-pass hepatic metabolism (43, 48).

The microemulsion matrix, reinforced with rosmarinic acid as a natural antioxidant, provides a protective microenvironment for labile vitamins. Vitamin C, while hydrophilic, is highly susceptible to oxidative degradation in aqueous solution. Encapsulation within the aqueous phase of a surfactant-stabilized system, combined with the antioxidant activity of rosmarinic acid, limits exposure to pro-oxidant factors (29, 32, 33). Similarly, the oil-water interface of the microemulsion droplets can protect vitamin D_3_ and vitamin E from acidic degradation in the stomach, reducing the risk of precipitation upon gastric pH transition (42, 43). The stability data (Section 3.4) confirm that the formulation maintains >90% vitamin integrity under recommended storage, suggesting that vitamin molecules remain intact and available for absorption at the time of consumption.

The PEG-based surfactants (Polysorbate 80 and PEG 400 monooleate) used in this formulation serve dual roles as emulsifiers and potential permeation enhancers. Polysorbate 80, in particular, has been shown to transiently modulate tight junctions in intestinal epithelial cells and inhibit P-glycoprotein efflux pumps, thereby increasing paracellular and transcellular transport of co-administered compounds (46, 47). While these effects are well-documented for drug delivery, similar principles apply to nutrient absorption. The presence of these surfactants at the oil-water interface, combined with the nano-emulsion structure, may therefore contribute to enhanced absorption of both hydrophilic and lipophilic vitamins (48).

The Comparative bioavailability studies with other vitamin-fortified beverages and conventional supplements would be required to quantify the advantage of this nano-emulsion platform. However, the literature supports the premise that lipid-based nano-formulations can significantly improve the oral bioavailability of fat-soluble vitamins. For example, nano-emulsions of vitamin D_3_ have demonstrated 2- to 4-fold increases in area under the curve (AUC) compared to conventional oil-based formulations in preclinical models (44, 45). Similarly, co-encapsulation of multiple vitamins within a single nanostructured carrier may promote synergistic absorption and reduce inter-individual variability (48).

### 4.1 Rosmarinic acid as a dual-function bioactive: beyond preservation

The inclusion of rosmarinic acid in this formulation was initially intended to protect labile vitamins from oxidative degradation during storage. However, recent *in vivo* evidence from our group Salako et al. (59) demonstrates that rosmarinic acid exerts potent nephroprotection in a gentamicin-induced acute kidney injury model through at least three complementary mechanisms: (i) activation of the Nrf2/HO-1 antioxidant pathway (2.5-fold increase in nuclear Nrf2, 2.1-fold increase in HO-1, and upregulation of NQO1 and GCLC), (ii) suppression of NF-κB-mediated inflammation (61% reduction in nuclear NF-κB p65, with corresponding decreases in TNF-α, IL-1β, and IL-6), and (iii) preservation of mitochondrial membrane potential and reduction of oxidative DNA damage (58% decrease in 8-OHdG).

These findings have direct relevance to the intended use of our vitamin-fortified beverage. Chronic dehydration, particularly in hot climates, is associated with increased oxidative stress and low-grade inflammation in renal tissues (60). By incorporating rosmarinic acid at a concentration of 5 mg per 250 mL serving (equivalent to the effective oral dose in rats after allometric scaling), the beverage may provide passive chemoprotection against dehydration-induced renal injury, in addition to delivering essential vitamins. Moreover, the anti-inflammatory properties of rosmarinic acid (reduction in TNF-α and IL-6) align with the beverage's stated goal of immune support, complementing the immunomodulatory roles of vitamins C and D_3_.

It is important to note that these mechanistic data were obtained from an oral rosmarinic acid extract in a rat model, not from the Nano-emulsified beverage. However, the Nano-emulsion platform is expected to enhance rosmarinic acid's bioavailability compared to simple aqueous solutions (61), potentially improving its efficacy, which Maria et al. (62) fail to address due to their inability to establish the dosage ratio of Rosmarinic acid in the formulation of the nano-emulsion substance they based their study on. Future studies should directly assess whether the rosmarinic acid within our nano-emulsion retains its Nrf2-activating and NF-κB-suppressing activities after gastrointestinal transit.

## 5 Limitations and future outlooks

The current work does not include *in vivo* bioavailability studies, which represent the logical next step in translation. Future research should prioritize:

- Pharmacokinetic studies in healthy volunteers comparing the absorption kinetics of vitamins C, B_2_, D_3_, and E from this beverage vs. an equivalent conventional supplement (e.g., tablet or non-emulsified liquid) using validated LC-MS/MS methods
- Food effect studies to evaluate whether the nano-emulsion maintains consistent absorption regardless of prandial state
- Mechanistic investigations using *in vitro* models such as Caco-2 cell monolayers to assess transport mechanisms, permeability enhancement, and potential interactions between vitamins and surfactants
- Clinical outcome studies to evaluate functional endpoints such as immune response markers, hydration status, and cognitive performance in target populations (e.g., elderly, athletes, or individuals in high-heat environments)

## 6 Conclusions

This study successfully developed and comprehensively characterized a science-based protocol for producing a novel multi-vitamin fortified beverage designed to address both hydration and nutritional needs in the context of increasing global temperatures. The key accomplishment lies in the formulation of a stable, clear, and palatable beverage that combines both water-soluble (Vitamins C and B_2_) and fat-soluble (Vitamins D_3_ and E) vitamins—a notable challenge in beverage science.

The innovation of this work is 2-fold. First, the two-stage production method—creating a stabilized microemulsion concentrate using PEG-based surfactants and rosmarinic acid, followed by dilution into specially prepared water—proved effective in overcoming solubility and instability barriers. Second, the non-conventional sequential mixing process ensured preservation of vitamin integrity and promoted a homogeneous final product.

Comprehensive physicochemical characterization confirmed the formation of a stable microemulsion with a mean droplet diameter of 89.3 nm, a zeta potential of −32.4 mV, and a narrow polydispersity index of 0.187. HPLC-DAD analysis with full method validation confirmed vitamin identity and enabled accurate quantification (14).

The 12-week stability study under multiple storage conditions demonstrated that the formulation maintains >90% vitamin retention for 12 weeks under refrigeration and 8 weeks at room temperature when protected from light. Light exposure and elevated temperatures accelerate degradation, highlighting the importance of appropriate packaging and storage recommendations.

The resulting beverage, with a consumer-friendly pH of 6.0 and a pleasant taste profile, represents a practical, functional alternative to plain water. It is specifically designed to support populations in arid and high-heat regions, where dehydration risks are compounded by climate change and where access to varied nutrition may be limited.

While this study establishes a robust, validated formulation with comprehensive characterization, further work is essential to translate this formulation into a widely adoptable solution. Future research should prioritize clinical trials, pharmacokinetic studies, and scalability assessments.

In summary, this formulation offers a promising, science-driven approach to functional hydration, aligning nutritional supplementation with public health needs in an era of environmental change. It underscores the potential of integrated food science to create targeted dietary solutions that are both preventive and supportive of overall wellbeing.

## Data Availability

The original contributions presented in the study are included in the article/Supplementary material, further inquiries can be directed to the corresponding author.
