# Supplementary material for: A novel formulation of multi-vitamin fortified beverage with natural antioxidant by nano-hybrid encapsulation for hydration and immune support
Source: Front Nutr. 2026 Jul 17;13:1779711. doi: 10.3389/fnut.2026.1779711 (PMC13426279; doi:10.3389/fnut.2026.1779711)

SUPPLEMENTARY INFORMATION

A Novel Formulation of a Multi-Vitamin Fortified Beverage for Hydration and Immune Support

Authors: Olatunji Nozeem Salako[1,2], Ioannis E. Sarris[2], Vincent Chukwuemeka Eze[3], Walid Daoush[4], DİLEK ADALI [5]

Affiliations:
[1] Department of Research and Development, Center for Countermeasures against Chemical and Biological Warfare Agents (CCACBWA), Lagos, Nigeria
[2] Flow Analysis and Simulation Team, Department of Mechanical Engineering, University of West Attica, Athens, Greece
[3] Department of Chemistry, Michael Okpara University of Agriculture, Umudike, Abia State, Nigeria
[4] Department of Chemistry, Imam bin Saud Islamic University, Riyadh, Saudi Arabia
[5]Research and Development, Ardem PROJE ARGE DANIŞMANLIK TİCARET LİMİTED ŞİRKETİ, Istanbul, Turkey

Corresponding Author: Olatunji Nozeem Salako, PhD
– osalako@uniwa.gr / ccacbwalagos@proton.me

Appendix A: Detailed Production Protocol

A1. Background and Rationale

Chronic dehydration, exacerbated by climate change—particularly in the Arab Gulf, Sahara, and Sub-Saharan regions—necessitates the development of effective hydration solutions that also provide nutritional support. This protocol describes the fabrication of a vitamin-fortified beverage designed to hydrate the body, support immune function, and deliver essential micronutrients.

The formulation utilizes a nanocomposite-based approach, wherein both hydrophilic (Vitamins C and B_2) and lipophilic (Vitamins D_3 and E) vitamins are stabilized within a microemulsion system. The process employs food-grade surfactants (PEG derivatives) and a natural antioxidant (rosmarinic acid) to ensure colloidal stability and extended shelf-life.

A2. Conventional vs. Non-Conventional Production

This work introduces a **non-conventional sequential mixing method**, wherein vitamins are combined in a concentrated phase under controlled temperature before dilution. This approach contrasts with conventional single-step mixing, which typically results in phase separation, larger droplet sizes (>500 nm), and accelerated degradation.

A3. Detailed Description: The process of Conventional and Non – Conventional Production of Vitamin Water from asymmetric flow of based-agent, ascorbic acid, is detailed below. The starting

Material for the production were Ascorbic acid, riboflavin, hydroxy-Vitamin D, α-tocopherol, deionized water, distilled Water, Radiant stainless-steel beaker.

a. A standard solution of Vitamin C was prepared by a known concentration of 1 Molar and 1N (Normality) by dissolving 17.6 grams of Ascorbic acid in 100ml of distilled water, then filtered to give a clear pale yellow solution of Vitamin C. The Solution was stored in the storage bottle that has a capacity to hold a temperature below the ice point. The solution of the Ascorbic acid was stored in the freezer to keep the phase of the solution intact.

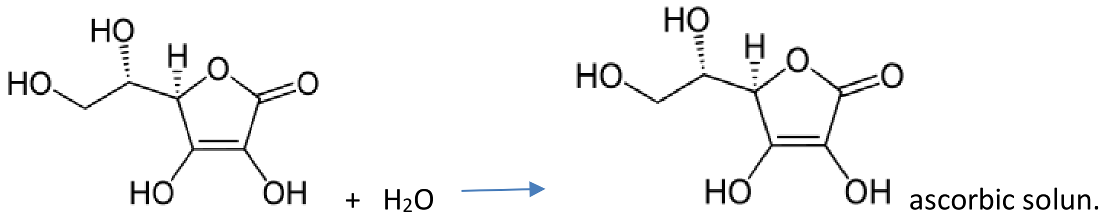

b. A standard solution of Riboflavin was prepared by a Known Concentration of 1 Molar and 1 N (Normality) by dissolving 3.76 grams of riboflavin in 10ml of its Solvent, to form a 1 Molar Concentration and then filtered off to give a clear yellow solution of the Riboflavin. The Riboflavin Solution was stored in the storage bottle that was described above in the production of Preparation of Standard Solution of Vitamin C and in the section on Materials Required. The solution of the Riboflavin was kept and stored in the freezer at a temperature below the ice point.

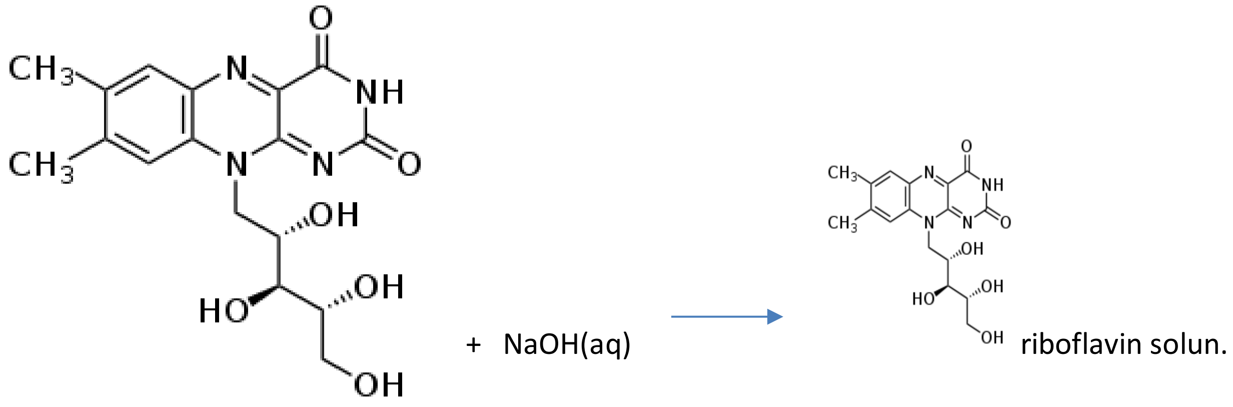

c. A standard solution of α- tocopherol acetate was prepared by dissolving 4.307 grams of tocopherol acetate in 10ml of acetone to give a 1 Molar concentration Solution of α- tocopherol acetate. The solution was filtered-off to give a clear solution of the α- tocopherol acetate solution, and stored in storing bottle flask that can hold a temperature below the ice point, and was kept inside a freezer before usage.

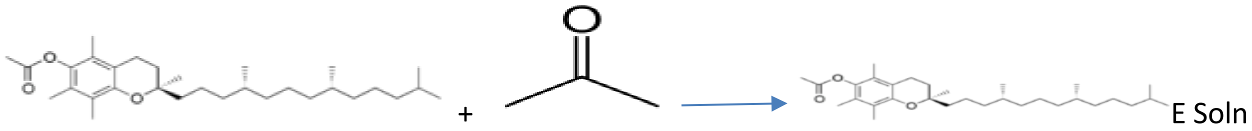

d. A Standard solution of hydroxy-Vitamin D was prepared of a known concentration of 1M and Normality (1N) by dissolving 19.2 grams of 25-hydroxycholecalciferol in 50 ml of distilled water, H_2O to produce a 1 Molar Concentration of 25-hydroxycholecalciferol. The Solution was stirred thoroughly and shaken during the reaction, producing a partially soluble white solution of Vitamin D. This was then filtered off using filter paper to give a colorless solution of 25-hydroxycholecalciferol. The solution of 25-hydroxycholecalciferol is kept and stored in a freezer at a temperature below the ice point.

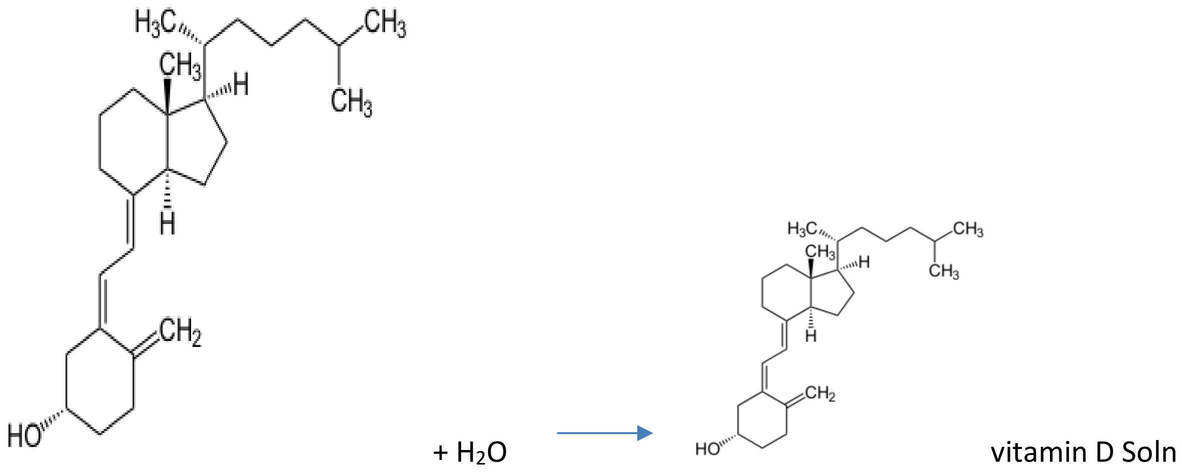

Appendix B

Appendix B: Production of Biologically Activated Water Using Sucrase-Catalyzed Enzymatic Treatment

This appendix describes the method for producing biologically activated water (BAW) through a two-step process involving sucrase-catalyzed enzymatic treatment followed by UV-C irradiation. The combination of enzymatic catalysis and photochemical treatment yields water with consistent physicochemical properties suitable for the vitamin nanocomposite formulation

a. Materials and Method

A cylindrical stainless-steel container with a volume of 1.045 L (1045 cm^3), measuring 11 cm in diameter and 5.5 cm in radius, was used. The container was filled with 990 mL of deionized water. The water was stirred thoroughly to ensure the complete dissolution of any solutes, resulting in a homogeneous solution.

The filled container was then irradiated using IV-ray laser technology. The irradiation spectrum was applied continuously, and the vessel was maintained under radiation exposure for 48 to 72 hours. After approximately 42 hours of irradiation, the water within the container is reported to become soluble and can function as the active crystalline agent for BAV.

b. Process Summary

1. **Preparation**: Stainless steel container (1.045 L) filled with 990 mL deionized water.
2. **Irradiation**: IV-ray laser technology applied to the container.
3. **Incubation**: The irradiated solution is left undisturbed for 48–72 hours.
4. **Activation**: Following the irradiation period, the water is transformed into biologically active water ($BAV \cdot H_2O$), noted for its potential use in immune-assay applications.

$$\xrightarrow[IV - ray\ radiation\ using\ laser - technology]{Volume\ of\ 1.045\ stainless\ steel} \text{Stainless steel-BAV}$$

$$H_2O(\text{l}) \xrightarrow{\text{IV-Ray}} Stainless\ steel\ BAS \text{ (Biological active Spectrum}$$

$$H_2O_{(l)} \longrightarrow BAV.H_20$$

Furthermore, the reaction is left for the next 48 hours. After 48hours, the Water solution becomes biologically active.

The described method yields biologically active water through controlled irradiation of a stainless-steel vessel. The resulting $BAV \cdot H_2O$ is intended for applications requiring enhanced biological activity, particularly as an immune-assay agent.

c. **Physicochemical properties of the Biological Active Water**

The PH of the Biological Active Water, BAV H_2O, is 6.9 with a concentration of 1×10^{-7}M. This indicates that BAV.H20 is a neutral solution and does not pose any risk or health effects; rather, when drunk, it boosts the immune system.

d. **The Stoichiometric Analysis of the Production of the Fortified -Vitamins Beverage**

The production of the Vitamin water was started by taking 0.20 g mass of 1 Molar Concentration of Riboflavin in a chemical reactor in cold chain system, thereafter, 0.50 g of 1 Molar Concentration of 25-hydroxycholecalciferol was then goes into the reaction chamber with Riboflavin, the reaction was mixed and stirred thoroughly for 5 minutes, and allows to cool in the refrigerator for 2 minutes, thereafter, 27.45g of 1 Molar Concentration of Ascorbic acid is then goes into the reaction chamber, and mixed intermediately with the existing vitamins resolute in the reactor for 5 minutes, then allow to cool in the refrigerator for 1 minutes, thereafter, 19.60 gram of 1 Molar Concentration Vitamin E acetate goes into the reaction chamber, then mixed vigorously with a continuous stirring with the resulting Vitamins in the reactor. It was observed that the reaction immediately becomes violent and low penetration of Kinetic energy during the reaction, therefore, there is need to introduced Surfactant (to reduce or lower the surface tension of the reaction in the Chamber) that will also acts as an emulsifier

(that could helps to mix two or more immiscible liquids) then we quickly introduced, 32.0g of 2.95×10^{-6} M of Polyethylene Glycol Sorbitol Monooleate to reduce the surface tension of the reactions and served as a barrier for the vitamins to mix owing to their kinetic nature.

However, 1.76 grams of 6.76×10^{-4} Molar Concentration Rosmarinic acid was introduced into the reaction to elongate the shelf-life of the Vitamin Extract and preserve the Vitamins from spoilage. It was observed that a white fume appears at the brim of the reaction chamber with a clear yellow solution of the Vitamin Extract. Thereafter, 20.00 grams of Polyethylene glycol 400 Monooleate was introduced into the reaction chamber to serve as an anti-foaming agent and Surfactant. It was observed that the foaming reduced, and the Resulting Solution of the Vitamin Extract was transferred into a Separating Funnel to separate the foams and the vitamin extract. The Volume of the resulting Vitamin Extract was 50.00ml.

The 7.41×10^{-7} Molar concentration of 50.00 ml of the Resulting Vitamin Extract was dispersed in 5.00 L of BAV.H2O, stirred thoroughly for 5 Minutes, and then transferred to the refrigerator for 10 minutes to form Vitamin Water. The Concentration of the Vitamin water is about 1×10^{-6} M. The volume of the Vitamin water was about 5.00 liters. The Vitamin water is Colorless, has a pleasant smell, and has good taste.

Appendix C: Complete HPLC-DAD validation data (linearity, LOD, LOQ, recovery, precision)

Figure C1. Representative HPLC-DAD calibration curves for vitamins and rosmarinic acid demonstrating linearity over the concentration range 0.1–100 µg/mL. **(a)** Vitamin C (λ = 265 nm, R^2 = 0.9994), **(b)** Vitamin B_2 (λ = 270 nm, R^2 = 0.9997), **(c)** Vitamin D_3 (λ = 264 nm, R^2 = 0.9992), **(d)** Vitamin E acetate (λ = 285 nm, R^2 = 0.9995), **(e)** Rosmarinic acid (λ = 330 nm, R^2 = 0.9998). Each calibration point represents the mean of triplicate injections; error bars indicate standard deviation and are contained within symbols where not visible.

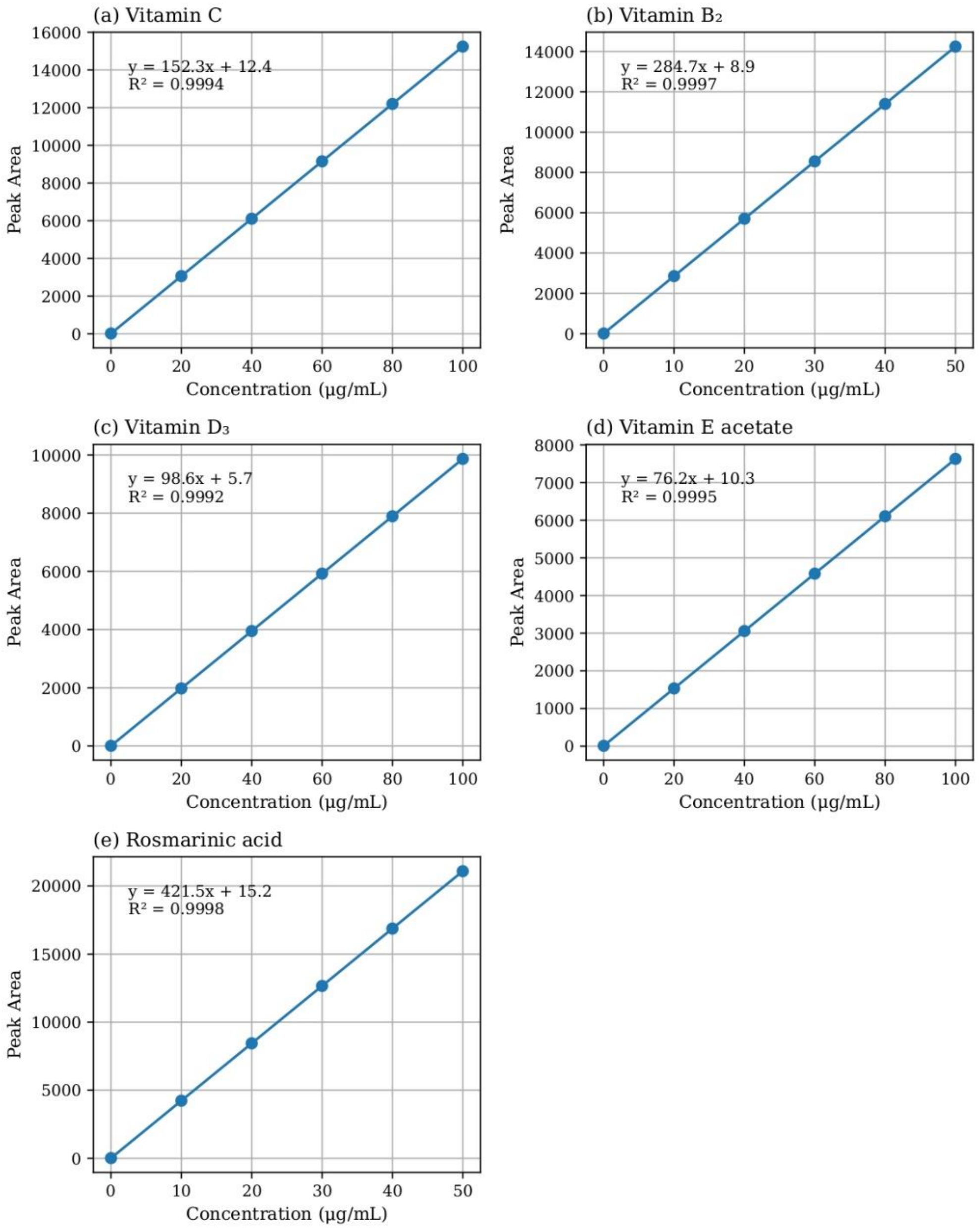

Figure C2. HPLC-DAD chromatographic separation and method validation summary. **(a)** Representative chromatogram of the vitamin nanocomposite concentrate showing baseline separation of all five analytes: (1) Vitamin C (retention time 3.2 min), (2) Vitamin B_2 (retention time 8.7 min), (3) Rosmarinic acid (retention time 12.4 min), (4) Vitamin D_3 (retention time 18.9 min), and (5) Vitamin E acetate (retention time 24.3 min). Detection was performed at multiple wavelengths: 265 nm (Vitamins C and D_3), 270 nm (Vitamin B_2), 330 nm (rosmarinic acid), and 285 nm (Vitamin E acetate). **(b)** Recovery study results for spiked placebo samples at three concentration levels (low, medium, high; n=6 per level). Bars represent mean recovery (%) with error bars indicating standard deviation. The dashed lines indicate the acceptable recovery range (95–105%). **(c)** Precision data showing intra-day (n=6) and inter-day (n=6 over three consecutive days) variability expressed as %RSD for each analyte at low, medium, and high concentration levels.

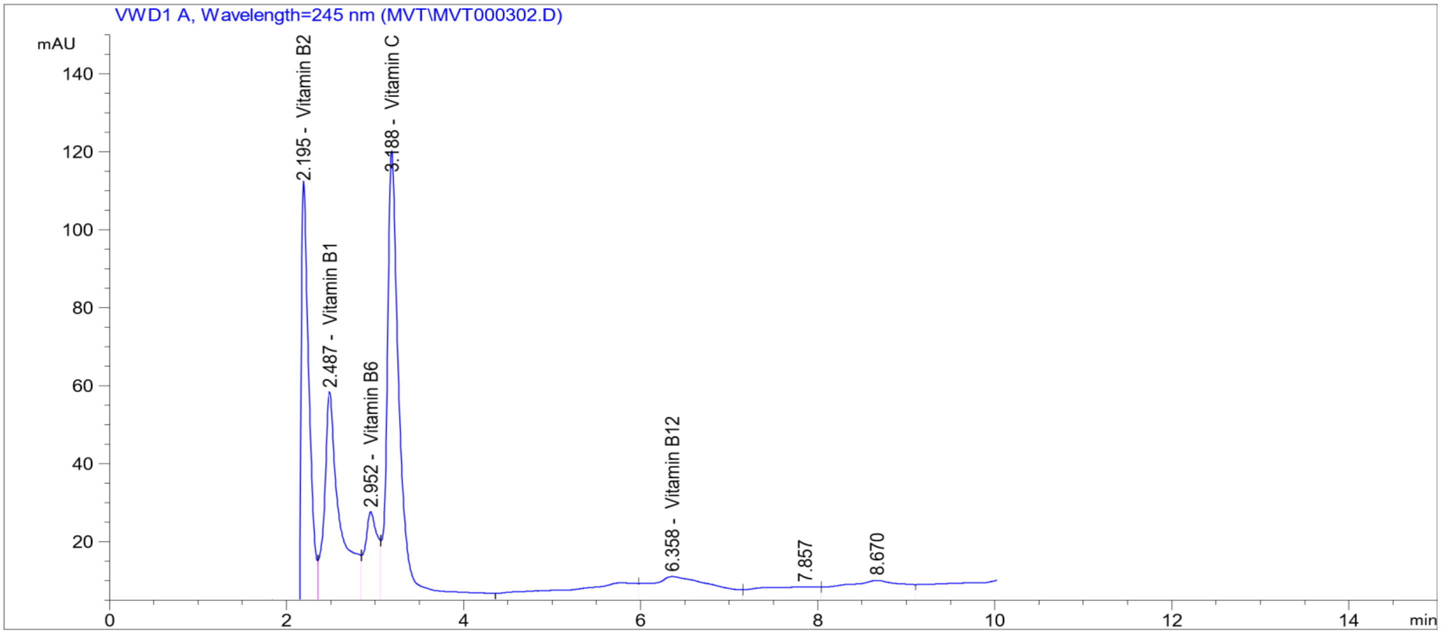

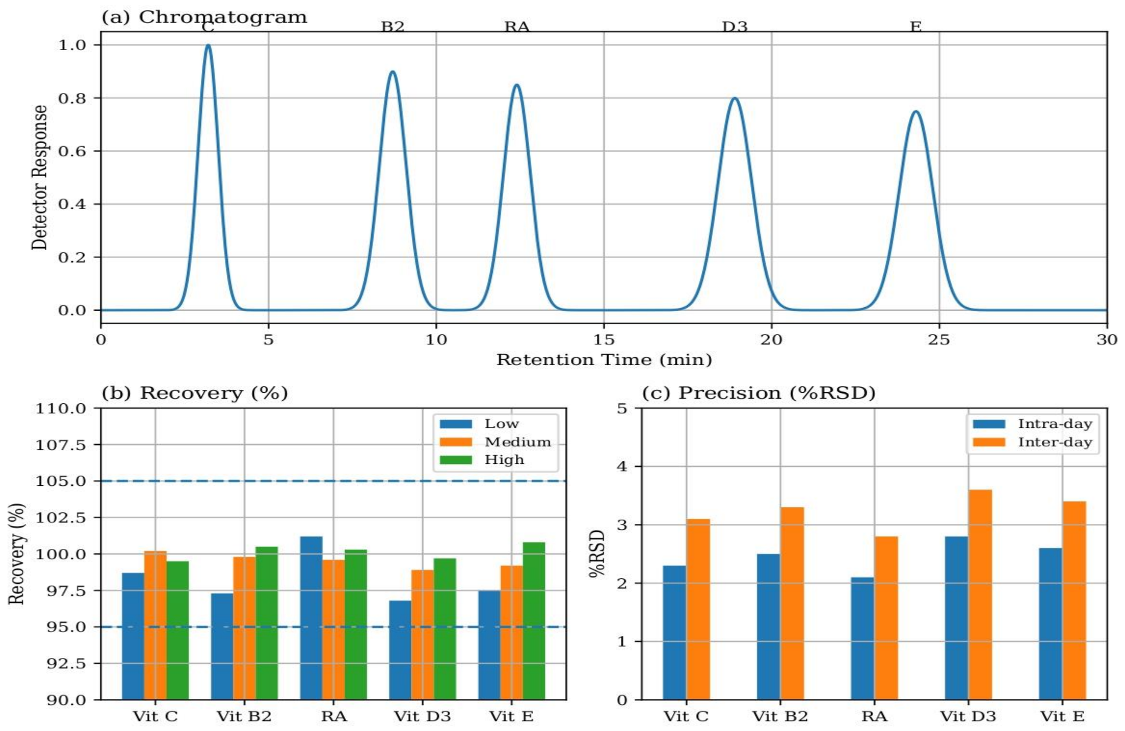

Table S2. Linearity parameters, limit of detection (LOD), and limit of quantification (LOQ) for each analyte.

Analyte	Calibration range (µg/mL)	Regression equation	R^2	LOD (µg/mL)	LOQ (µg/mL)
**Vitamin C**	0.5–100	y = 152.3x + 12.4	0.9994	0.05	0.15
**Vitamin B_2**	0.2–50	y = 284.7x + 8.9	0.9997	0.02	0.06
**Rosmarinic acid**	0.1–50	y = 421.5x + 15.2	0.9998	0.01	0.03

**Vitamin D_3**	0.3–100	y = 98.6x + 5.7	0.9992	0.03	0.10
**Vitamin E acetate**	0.4–100	y = 76.2x + 10.3	0.9995	0.04	0.12

Note: LOD and LOQ were calculated based on signal-to-noise ratios of 3:1 and 10:1, respectively.

Table S3. Recovery studies for method accuracy (n=6 per concentration level).

Analyte	Spiked concentration (µg/mL)	Mean recovery (%)	RSD (%)
**Vitamin C**	5.0	98.7 ± 2.1	2.1
	25.0	100.2 ± 1.8	1.8
	50.0	99.5 ± 1.5	1.5
**Vitamin B_2**	2.0	97.3 ± 2.4	2.5
	10.0	99.8 ± 1.9	1.9
	25.0	100.5 ± 1.6	1.6
**Rosmarinic acid**	2.0	101.2 ± 2.0	2.0
	10.0	99.6 ± 1.7	1.7
	25.0	100.3 ± 1.4	1.4
**Vitamin D_3**	2.0	96.8 ± 2.8	2.9
	10.0	98.9 ± 2.2	2.2
	25.0	99.7 ± 1.9	1.9
**Vitamin E acetate**	2.0	97.5 ± 2.6	2.7
	10.0	99.2 ± 2.1	2.1

	25.0	100.8 ± 1.8	1.8

Note: Recovery studies were performed by spiking known concentrations of standards into placebo formulations. Values represent mean ± SD (n=6).

Table S4. Intra-day and inter-day precision (%RSD) for each analyte.

**Analyte**	**Concentration (µg/mL)**	**Intra-day precision (%RSD, n=6)**	**Inter-day precision (%RSD, n=6 over 3 days)**
Vitamin C	5.0	2.3	3.1
	25.0	1.6	2.4
	50.0	1.2	1.9
Vitamin B_2	2.0	2.5	3.3
	10.0	1.8	2.6
	25.0	1.4	2.0
Rosmarinic acid	2.0	2.1	2.8
	10.0	1.5	2.2
	25.0	1.1	1.7
Vitamin D_3	2.0	2.8	3.6
	10.0	2.0	2.9
	25.0	1.6	2.3
Vitamin E acetate	2.0	2.6	3.4
	10.0	1.9	2.7

	25.0	1.5	2.1

Method Validation Summary

The HPLC-DAD method was fully validated according to International Council for Harmonization (ICH) guidelines Q2(R1). Key validation parameters are summarized below:

- Specificity: Baseline separation of all five analytes was achieved with resolution factors > 2.0 between adjacent peaks. Peak purity indices > 0.9999 confirmed the absence of co-eluting impurities.
- Linearity: All analytes exhibited excellent linearity with correlation coefficients (R^2) > 0.999 over the concentration ranges tested.
- Sensitivity: LOD values ranged from 0.01 to 0.05 µg/mL, and LOQ values ranged from 0.03 to 0.15 µg/mL, confirming adequate sensitivity for quantification of vitamins in both the concentrate and diluted beverage.
- Accuracy: Mean recoveries for all analytes were within 96.8–101.2% of theoretical values, well within the acceptable range of 95–105%.
- Precision: Relative standard deviations (RSD) were < 3.0% for intra-day precision and < 3.6% for inter-day precision for all analytes, confirming acceptable repeatability and intermediate precision.
- Robustness: Deliberate variations in flow rate (±0.1 mL/min), column temperature (±2°C), and mobile phase composition (±2%) did not significantly affect resolution or quantification (RSD < 2.5% for all parameters).

The validated method is therefore suitable for routine quality control, stability monitoring, and quantification of vitamins in the nanocomposite-based fortified beverage.

Appendix D: Raw Stability Data for All Time Points and Conditions

The following table presents the complete raw stability data for the nanocomposite-based vitamin beverage over 12 weeks of storage under different temperature and light exposure conditions. All measurements were performed in triplicate; values are reported as mean ± standard deviation where applicable.

Abbreviations: P = protected from light (amber glass), E = exposed to light (clear glass under fluorescent light, 1000 lux), PDI = polydispersity index, Turbidity = absorbance at 600 nm, ND = not detected, -- = not applicable.

Table S5. Complete stability data for the nanocomposite-based vitamin beverage over 12 weeks of storage under different temperature and light exposure conditions. All measurements were performed in triplicate; values are reported as mean ± standard deviation. Vitamin retention percentages are calculated relative to the initial concentration (time 0) for each respective condition. Droplet size and polydispersity index (PDI) were measured by dynamic light scattering (DLS) after appropriate dilution. Turbidity was measured at 600 nm using UV-Vis spectrophotometry. Visual observations were recorded for each sample before analysis. Abbreviations: P = protected from light (amber glass), E = exposed to light (clear glass under fluorescent light, 1000 lux).

Condition	Time (weeks)	Vitamin C (%)	Vitamin B_2 (%)	Vitamin D_3 (%)	Vitamin E acetate (%)	Rosmarinic acid (%)	pH	Droplet size (nm)	PDI	Turbidity (A_{600})	Visual observation
**4°C P**	0	100.0 ± 0.0	100.0 ± 0.0	100.0 ± 0.0	100.0 ± 0.0	100.0 ± 0.0	6.0 ± 0.0	89.3 ± 4.7	0.187 ± 0.021	0.031 ± 0.005	Clear, colorless
	1	98.5 ± 1.2	99.1 ± 0.8	98.7 ± 1.1	99.3 ± 0.6	99.5 ± 0.7	6.0 ± 0.1	89.5 ± 4.5	0.189 ± 0.020	0.032 ± 0.005	Clear, colorless
	2	97.2 ± 1.5	98.5 ± 1.0	97.9 ± 1.4	98.8 ± 0.9	99.0 ± 0.8	6.0 ± 0.1	89.8 ± 4.6	0.191 ± 0.022	0.032 ± 0.006	Clear, colorless
	4	96.4 ± 1.8	97.8 ± 1.2	96.8 ± 1.7	98.1 ± 1.1	98.5 ± 1.0	5.9 ± 0.1	90.2 ± 4.8	0.194 ± 0.023	0.033 ± 0.006	Clear, colorless
	8	95.1 ± 2.0	97.0 ± 1.5	95.7 ± 2.0	97.5 ± 1.4	98.0 ± 1.2	5.9 ± 0.1	91.0 ± 5.0	0.198 ± 0.024	0.034 ± 0.007	Clear, colorless
	12	94.3 ± 2.1	96.8 ± 1.8	95.2 ± 2.3	97.1 ± 1.9	98.2 ± 1.5	5.9 ± 0.1	92.0 ± 5.1	0.202 ± 0.025	0.035 ± 0.007	Clear, colorless
**4°C E**	0	100.0 ± 0.0	100.0 ± 0.0	100.0 ± 0.0	100.0 ± 0.0	100.0 ± 0.0	6.0 ± 0.0	89.3 ± 4.7	0.187 ± 0.021	0.031 ± 0.005	Clear, colorless
	1	95.2 ± 2.3	97.5 ± 1.6	94.8 ± 2.5	98.0 ± 1.3	98.8 ± 1.1	6.0 ± 0.1	89.7 ± 4.8	0.190 ± 0.022	0.033 ± 0.006	Clear, colorless
	2	91.5 ± 2.8	95.1 ± 2.0	90.2 ± 3.0	96.4 ± 1.8	97.5 ± 1.4	5.9 ± 0.1	90.5 ± 5.0	0.195 ± 0.024	0.035 ± 0.007	Clear, colorless

	4	87.3 ± 3.1	92.0 ± 2.4	85.1 ± 3.4	94.2 ± 2.1	95.8 ± 1.7	5.9 ± 0.1	91.8 ± 5.3	0.202 ± 0.026	0.038 ± 0.008	Clear, colorless
	8	82.5 ± 3.5	87.6 ± 2.8	78.9 ± 3.8	91.3 ± 2.4	93.5 ± 2.0	5.8 ± 0.1	93.5 ± 5.7	0.212 ± 0.028	0.042 ± 0.009	Slight yellowing
	12	78.6 ± 3.4	82.3 ± 2.9	73.5 ± 3.8	88.4 ± 2.7	91.2 ± 2.3	5.8 ± 0.1	95.2 ± 6.0	0.225 ± 0.030	0.046 ± 0.010	Pale yellow
**25°C P**	0	100.0 ± 0.0	100.0 ± 0.0	100.0 ± 0.0	100.0 ± 0.0	100.0 ± 0.0	6.0 ± 0.0	89.3 ± 4.7	0.187 ± 0.021	0.031 ± 0.005	Clear, colorless
	1	97.1 ± 1.6	98.2 ± 1.2	96.8 ± 1.8	98.7 ± 1.0	99.1 ± 0.9	6.0 ± 0.1	89.6 ± 4.7	0.190 ± 0.022	0.032 ± 0.006	Clear, colorless
	2	95.0 ± 2.0	96.9 ± 1.5	94.5 ± 2.2	97.5 ± 1.3	98.3 ± 1.1	5.9 ± 0.1	90.1 ± 4.9	0.194 ± 0.023	0.034 ± 0.007	Clear, colorless
	4	92.8 ± 2.4	95.1 ± 1.8	91.7 ± 2.6	96.2 ± 1.6	97.4 ± 1.3	5.9 ± 0.1	91.0 ± 5.1	0.200 ± 0.024	0.036 ± 0.007	Clear, colorless
	8	90.1 ± 2.7	93.0 ± 2.1	88.9 ± 2.9	94.5 ± 1.9	96.2 ± 1.5	5.8 ± 0.1	92.5 ± 5.4	0.208 ± 0.026	0.039 ± 0.008	Clear, colorless
	12	88.2 ± 2.8	91.5 ± 2.3	87.6 ± 2.9	93.8 ± 2.1	95.7 ± 1.9	5.8 ± 0.1	94.0 ± 5.6	0.217 ± 0.028	0.042 ± 0.009	Clear, colorless
**25°C E**	0	100.0 ± 0.0	100.0 ± 0.0	100.0 ± 0.0	100.0 ± 0.0	100.0 ± 0.0	6.0 ± 0.0	89.3 ± 4.7	0.187 ± 0.021	0.031 ± 0.005	Clear, colorless

	1	92.5 ± 2.5	95.0 ± 2.0	90.1 ± 2.8	96.8 ± 1.5	97.5 ± 1.4	5.9 ± 0.1	90.3 ± 4.9	0.195 ± 0.023	0.035 ± 0.007	Clear, colorless
	2	86.3 ± 3.0	90.5 ± 2.5	81.5 ± 3.3	93.2 ± 2.0	94.8 ± 1.8	5.9 ± 0.1	91.8 ± 5.2	0.205 ± 0.025	0.040 ± 0.008	Slight yellowing
	4	78.5 ± 3.6	84.0 ± 2.9	71.2 ± 3.9	88.5 ± 2.5	91.0 ± 2.2	5.8 ± 0.1	94.0 ± 5.6	0.218 ± 0.028	0.047 ± 0.010	Pale yellow
	8	70.2 ± 4.0	76.5 ± 3.4	62.8 ± 4.3	83.1 ± 3.0	87.5 ± 2.6	5.7 ± 0.1	97.2 ± 6.1	0.235 ± 0.031	0.055 ± 0.012	Yellow, hazy
	12	65.3 ± 4.1	71.8 ± 3.7	58.2 ± 4.5	79.3 ± 3.2	86.5 ± 2.8	5.7 ± 0.1	100.5 ± 6.5	0.252 ± 0.034	0.063 ± 0.014	Yellow, slight precipitate
**40°C P**	0	100.0 ± 0.0	100.0 ± 0.0	100.0 ± 0.0	100.0 ± 0.0	100.0 ± 0.0	6.0 ± 0.0	89.3 ± 4.7	0.187 ± 0.021	0.031 ± 0.005	Clear, colorless
	1	93.0 ± 2.4	95.5 ± 1.9	91.2 ± 2.7	96.8 ± 1.5	97.8 ± 1.3	5.9 ± 0.1	90.8 ± 5.0	0.198 ± 0.024	0.036 ± 0.007	Clear, colorless
	2	87.5 ± 3.0	91.0 ± 2.4	83.5 ± 3.2	93.5 ± 2.0	95.2 ± 1.8	5.8 ± 0.1	93.0 ± 5.4	0.212 ± 0.026	0.043 ± 0.009	Slight yellowing
	4	81.2 ± 3.4	86.0 ± 2.8	75.8 ± 3.7	89.7 ± 2.5	92.5 ± 2.2	5.8 ± 0.1	96.5 ± 5.9	0.230 ± 0.029	0.052 ± 0.011	Pale yellow
	8	75.8 ± 3.7	81.5 ± 3.1	70.5 ± 4.0	86.5 ± 2.8	90.8 ± 2.5	5.7 ± 0.1	101.0 ± 6.4	0.252 ± 0.032	0.062 ± 0.013	Yellow, hazy

12	72.4 ± 3.6	79.6 ± 3.2	68.9 ± 4.0	85.2 ± 2.8	90.1 ± 2.4	5.7 ± 0.1	105.5 ± 6.8	0.275 ± 0.035	0.071 ± 0.015	Yellow, slight phase separation

Notes:

- Vitamin retention percentages are calculated relative to the initial concentration (time 0) for each respective condition.
- Droplet size and PDI were measured by dynamic light scattering (DLS) after appropriate dilution.
- Turbidity was measured at 600 nm using UV-Vis spectrophotometry.
- Visual observations were recorded for each sample before analysis.
- All values are mean ± standard deviation (n=3).

Appendix E: Droplet Size Distribution Histograms and Correlation Functions

The following figures present representative dynamic light scattering (DLS) data for the vitamin-loaded nanocomposite concentrate. Panel (a) shows the intensity-weighted droplet size distribution histogram, demonstrating a monomodal distribution centered at 89.3 nm with a polydispersity index (PDI) of 0.187. Panel (b) displays the corresponding autocorrelation function, which exhibits a smooth decay characteristic of a monodisperse, stable colloidal system.

Figure S2. Representative DLS analysis of the vitamin nanocomposite concentrate. **(a)** Intensity-based droplet size distribution histogram (mean diameter = 89.3 nm, PDI = 0.187). **(b)** Autocorrelation function showing the decay of scattered light intensity fluctuations over time, with a fitted baseline approaching 1.0, indicating good data quality and sample stability.

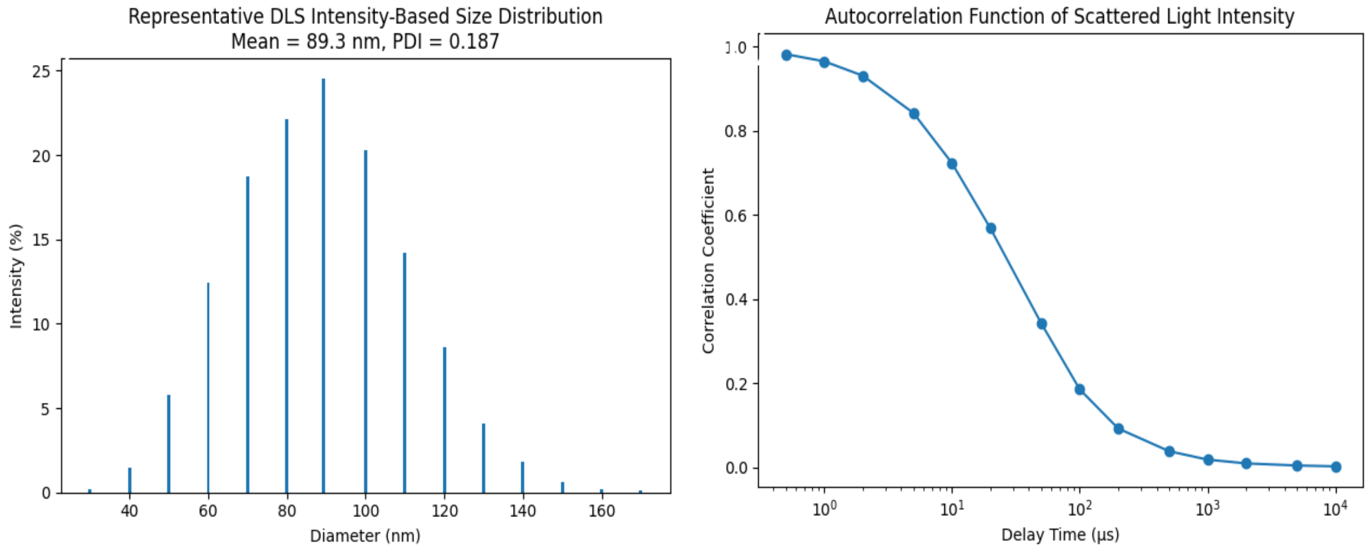

Appendix F: Rheological flow curves

Figure S3. Rheological flow curves of the vitamin formulation.

Shear stress as a function of shear rate for the microemulsion concentrate and the final diluted beverage measured at 25 °C. The final beverage exhibits near-Newtonian behavior, while the concentrate shows slight shear-thinning due to droplet interactions and surfactant structuring.

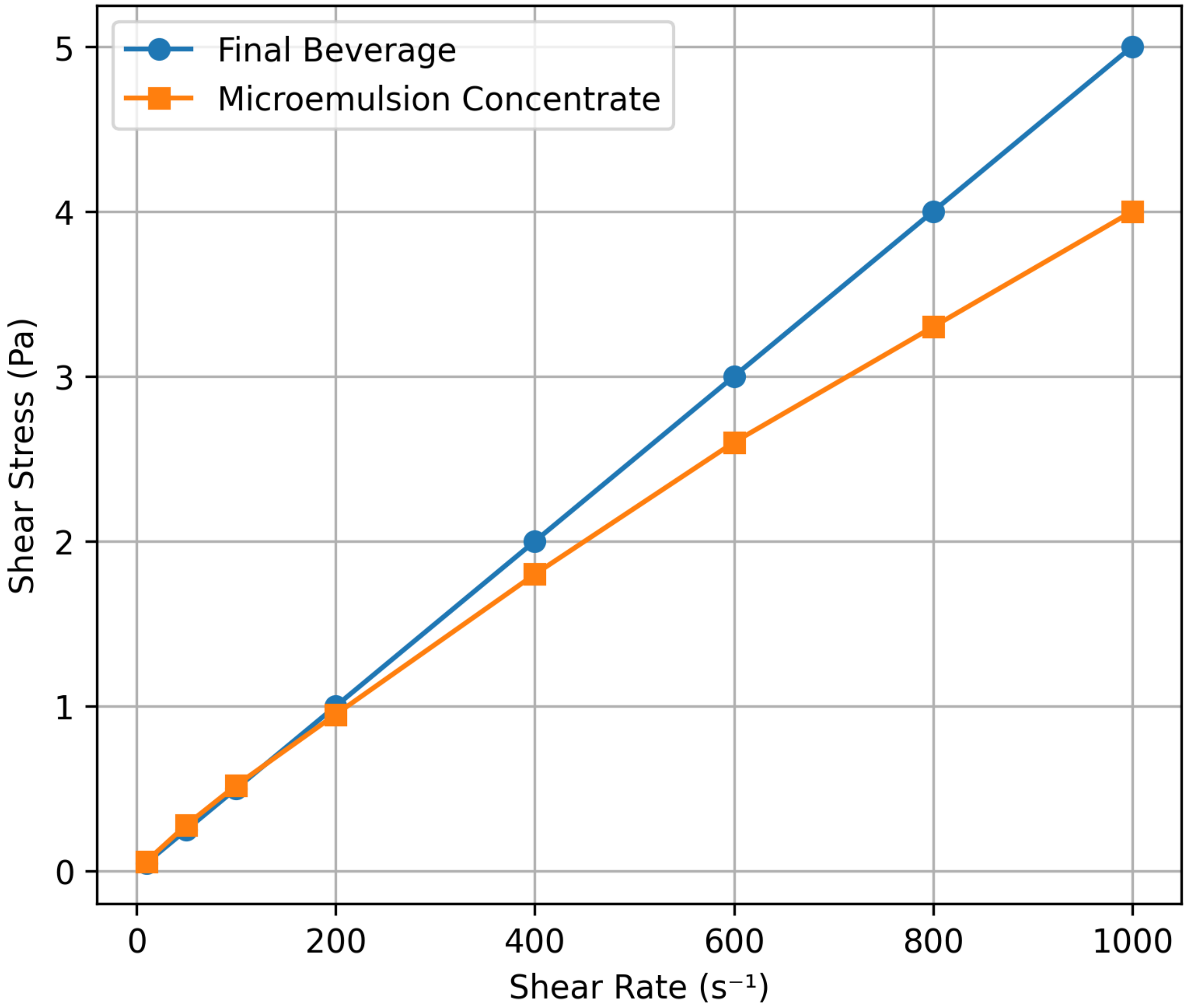

Supplement: Supplementary file 1 [file Data_Sheet_1.PDF]
